# The behaviour–performance continuum: how does individual variation in locomotor abilities relate to behaviour?

**DOI:** 10.1111/brv.70090

**Published:** 2025-10-28

**Authors:** Vincent Careau, Paul Agnani, Nicolas Bonin, Theodore Garland

**Affiliations:** ^1^ Department of Biology University of Ottawa 30 Marie Curie Ottawa ON K1N 6N5 Canada; ^2^ Department of Evolution, Ecology, and Organismal Biology University of California 900 University Ave Riverside CA 92521 USA

**Keywords:** evolutionary physiology, individual variation, lizards, locomotion, motivation, personality, treadmill

## Abstract

A series of terminological, technical, conceptual, and statistical challenges present themselves when trying to study correlations between measures of performance abilities (what an animal can do) and behavioural traits (what an animal chooses to do). We attempt to synthesise literature on this topic, with a specific focus on locomotor performance and behavioural traits measured with standardised tests. We argue that measures of forced performance (e.g. endurance on a motorised treadmill) and voluntary behaviour (e.g. wheel running) often fall along a continuum, sometimes grading into each other. On the performance end of the continuum, tests should measure what an animal *can do* when motivation is maximal and/or it is given no choice but to exert itself maximally. On the behavioural end of the continuum, tests should capture what animals *choose to do* of their own free volition, with no experimental attempt to affect motivation. Hence, performance tests attempt to eliminate variation in motivation by forcing all individuals to be maximally motivated, whereas variation in motivation is an inherent component of all behavioural tests. In some cases, however, differentiating between measures of performance *versus* behaviour can seem almost arbitrary. Moreover, individuals may consistently differ in how willing they are to ‘perform’ even when ‘forced’ to do so. We compiled studies reporting any association (covariation, correlation or linear regression) between putative measures of locomotor performance and behaviour in various taxa. The vast majority of those studies report phenotypic correlations, and only a handful have reported genetic correlations or explored potential correlated responses to selection on performance or behaviour. To our knowledge, this is the first global overview of how locomotor performance and behaviour covary in animals, and we believe that our synthesis will be useful to guide future research on locomotor performance and its relationship with other ecologically relevant traits. For example, we argue that a multi‐level (co)variance partitioning approach is necessary to gain insights into the importance of how motivation differs across levels (e.g. among‐ *versus* within‐individual variation, genetic *versus* environmental variation). Finally, we outline a multitude of compensation and co‐specialisation mechanisms that may occur between performance and behaviour, and propose future avenues for research that include selection and manipulative studies to help identify the role of correlational selection, individual experience, and predation detectability on the relationships between behaviour and performance.

‘Individualistic behavioural traits have long been overlooked, dismissed as either noise or maladaptive deviation to atypical situations.’ (Iguchi, Matsubara & Hakoyama, 2001, p. 351)

‘The major problem bedevilling studies of performance is motivation – for whatever reason, some individuals may not use their maximal capabilities in laboratory trials … Anyone who has conducted sprint trials with lizards is aware that some lizards in at least some trials will run at clearly sub‐maximal speeds.’ (Losos, Creer & Schulte, 2002, p. 58)

## I. INTRODUCTION

Workers studying lizards and snakes were among the first to recognise the importance of individual variation in locomotor performance abilities. For example, Bennett (1980) demonstrated repeatable among‐individual differences in laboratory sprint speeds of lizards, both among daily trials at a given temperature and among trials at different temperatures. Somewhat ironically, Bennett (1980) was titled ‘The thermal dependence of lizard behaviour’, perhaps in large part because he submitted to the journal *Animal Behaviour*. Arnold (1983) focused attention on such performance variation as a likely more direct target of natural selection, as compared with lower‐level (subordinate) traits that determine performance abilities, and proposed the use of path analysis to analyse such relationships.

Exactly how one should quantify performance abilities quickly became an issue. For example, Garland (1985) sought to measure maximal sprint speeds of *Ctenophorus nuchalis* lizards by chasing them along a racetrack lined with 12 sets of photocells. He conducted eight trials over two test days, and then analysed the fastest 0.5 m interval recorded in relation to sex, body size, and relative limb, and tail lengths. Although not stated in the paper, the rationale for analysing the single highest performance value observed for each individual was that (*i*) lower values likely represented trials in which the animal was not maximally motivated or perhaps stumbled, and (*ii*) maximal performance measures should best reflect differences in locomotor morphology and physiology. In similar fashion, Losos & Sinervo (1989) conducted multiple sprint speed trials along rods of different diameters with four species of *Anolis* lizards, then analysed the fastest 0.1 m interval on each rod for each individual. Jayne & Bennett (1990) quantified selection acting on individual variation in both speed and endurance in a natural population of garter snakes (*Thamnophis sirtalis*). They also obtained multiple measures of performance for each individual, but analysed mean performance, stating that ‘Because it is nearly impossible to determine the extent of either measurement error or behavioural variation *versus* physiological capacity, we used mean values to minimise the potential effects of aberrant response by the animals during performance tests.’ (Jayne & Bennett, 1990, p. 1208).

Thus, from the beginning, studies have varied in how they treat variation in repeated performance measurements made on a given individual. Most commonly, however, researchers have preferred to retain only the maximum value per individual (Adolph & Pickering, 2008; Careau & Wilson, 2017a). Those ‘personal best’ scores were retained because any reductions in performance output were presumably due to lower motivation, measurement error or other difficulties (e.g. see Losos & Sinervo, 1989) and hence not reflective of maximal performance abilities, which are ultimately determined by lower‐level traits (e.g. organ size, muscle fibre types) (e.g. see fig. 11.4 in Foster *et al*., 2015). In addition, a measure of maximal performance will set the upper limit to behaviours that use that particular aspect of performance. And, as has been noted, with multiple different types of measures, one can conceive of both a ‘performance space’ (Bennett, 1989) and a ‘behaviour space’ (Losos, 1990), analogous to the ‘morphospace’ originally discussed by palaeontologists (e.g. Raup & Michelson, 1965; Schindel, 2007). How traits in these different domains relate to each other, which is part of the ‘genotype‐to‐phenotype map’, constitutes one of the great challenges of modern biology (e.g. see Ferry & Higham, 2022; Mykles *et al*., 2010; Padilla *et al*., 2014).

**Fig. 1:**
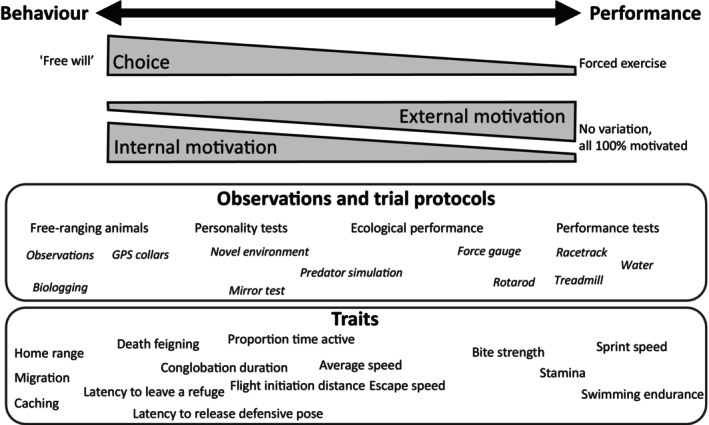
The behaviour–performance continuum, showing how different observation and trial protocols are used to measure various behavioural and performance traits, ordered along a continuum of decreasing behavioural choice and internal/external motivation. For example, to the left, animals have the choice to move freely (or not) and express their behaviour as they are internally motivated to do so. To the right, however, animals are given no choice but must conduct a single task at 100% of their capacity as they are prompted to do so by external sources of motivation [e.g. adverse stimuli such as electric shocks, frightening noises, being probed, being chased, etc., but in some cases by positive rewards (e.g. Harris & Steudel, 2002)]. When a free‐ranging animal encounters a predator, this external source of motivation provides information on its ecological performance, but the animal still has the choice to flee at any speed, remain motionless, or attack, such that there is a relatively large component of internal motivation in the measurements. When an animal is introduced into a personality test, it still has the choice to behave in particular ways, but that choice must be expressed within the structure of the test, which is much simplified compared to the natural environment (which is why personality tests capture traits that are to the right of the behavioural end of the continuum).

In the early 2000s, such neighbouring fields as behavioural ecology (Sih *et al*., 2004b; Wilson *et al*., 1994), endocrinology (Williams, 2008), and energetics (Bennett, 1987; Careau *et al*., 2008) started to embrace the full multi‐level variability inherent to complex organismal traits (e.g. variation within *versus* among individuals). To help make connections among these closely related fields, and to further our understanding of the multivariate evolution of performance, here we review the literature to explore the potential linkages between locomotor performance and animal behaviour. Although some of the links between performance and behaviour have been compiled recently as part of a larger review that also included metabolic traits (see Wu & Seebacher, 2022), the present review pays particular attention to how performance measurements are confounded with behaviour to various degrees, and how those terms are sometimes even used interchangeably. As a solution, we propose a behaviour–performance continuum (Fig. 1), along which performance and behavioural traits can be positioned depending on the amount of choice given to the animal and the relative influence of internal *versus* external motivation in the measured output. Finally, we highlight some of the insights that can be gained by applying a (co)variance partitioning approach to test for performance–behaviour relations.

### (1) Locomotor performance

Animal performance, defined as the ability of an organism to perform a task when maximally motivated (e.g. Careau & Garland, 2012; Garland & Losos, 1994), represents one of the main potential targets of natural selection (Arnold, 1983; Storz *et al*., 2015). Because of clear potential links to Darwinian fitness, studies of performance have made important contributions to the fields of organismal and evolutionary biology (Husak *et al*., 2006; Irschick *et al*., 2008; Matthews, Dial & Lauder, 2023; Miles, 2004). Performance abilities can affect (constrain) multiple daily tasks of an animal in its natural habitat, including foraging (Miles, Losos & Irschick, 2007), escaping from predators (Domenici, 2010), defending a territory (Lees *et al*., 2012; Peterson & Husak, 2006), traversing the home range (Singleton & Garland, 2018), acquiring mates (Pough, 1989), and fighting conspecifics (Husak & Fox, 2008; Lailvaux *et al*., 2004; Lappin & Husak, 2005). The list of possible whole‐organism performance traits is long, including maximum acceleration, sprint‐running speed, endurance, agility, and manoeuvrability in terrestrial animals (Bonine & Garland, 1999; Da Silva *et al*., 2014; Djawdan, 1993; Garland, 1999; Huey & Hertz, 1982; Tulli, Abdala & Cruz, 2012; Wilson *et al*., 2013; Wynn *et al*., 2015), various aspects of aquatic locomotion (Ghalambor, Walker & Reznick, 2003; Peterman & Ritterbush, 2022), and flying abilities in aerial animals (Dakin *et al*., 2018; Ellington, 1991).

Performance measures are not limited to aspects of locomotion, and may include such traits as the largest prey item that a snake can subdue or swallow (Arnold, 1983; Gartner & Greene, 2008). Moreover, some important performance traits involve only part of the organism, such as maximum bite force (Herrel, De Grauw & Lemos‐Espinal, 2001) or grip strength (Andrew *et al*., 2020; Berberi & Careau, 2019; Castro & Kuang, 2017). Thus, it is important to use operational definitions of performance traits, ideally tied to specific tests designed according to the species' behavioural ecology (e.g. foraging mode, anti‐predator strategies, intra‐sexual competition) and typical habitat or substrate (e.g. arboreal *versus* terrestrial). We note that Arnold (1983) emphasised that performance measures should be ‘ecologically relevant’, but we think this is a separate issue from the definition of performance. To be clear, performance does not need to be ecologically relevant to be definable, measurable, and verifiable as such. For example, one can measure many aspects of human performance (e.g. discus throw, shotput, shooting) that are likely not ‘ecologically relevant’ *per se*.

To keep our review focussed, we restricted our literature search to *locomotor* performance traits, but we consider other performance traits (e.g. bite force, grip strength, etc.) when discussing general challenges associated with measuring performance (e.g. motivation: see Section I.5). The maximal rate of oxygen during forced exercise (*V*O_2max_), usually on a motorised treadmill, is arguably a key aspect of performance (Dlugosz *et al*., 2013; Khan *et al*., 2024), but is excluded herein to help keep our review focused on movement. Moreover, we primarily discuss locomotor performance in rodents and squamates, as this is where our expertise lies, but our literature review nevertheless includes studies on a wide variety of organisms (e.g. insects, spiders, fish, amphibians).

**Fig. 2:**
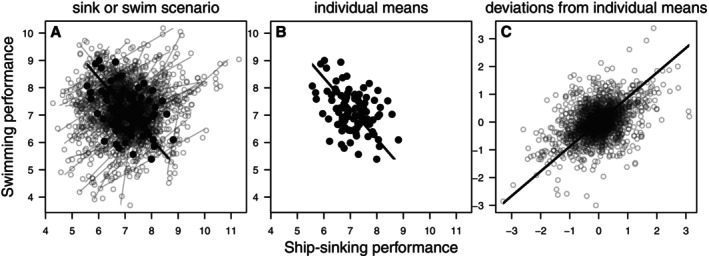
The sink‐or‐swim scenario. (A) Swimming performance as a function of ship‐sinking performance in a hypothetical population of legendary giant squid (Kraken) that were sampled 20 times throughout their life for their swimming and ship‐sinking performance (data from Careau & Wilson, 2017a). Solid black dots indicate mean values for each individual and the thick line illustrates the negative reduced major axis (RMA) regression at the among‐individual level, whereas thin grey lines show separate RMA regressions for each individual (most are positive). (B) Individual means illustrate how the two traits were part of a trade‐off at the among‐individual level, presumably due to consistent morphological or physiological differences (e.g. a slender body is good for swimming speed, but a wider body confers strength needed to sink ships). (C) Deviations from individual means illustrate within‐individual correlated phenotypic plasticity in response to an unknown factor, which could be water temperature, age, or training status. Careau & Wilson (2017a) suggested that this ‘sink or swim’ scenario likely applies to performance trade‐offs, because individuals consistently differ in morphology (e.g. tentacle length) or physiology (e.g. muscle contractile speed) such that, on average, strong individuals are slower, but whenever individuals train to increase their overall physical condition, their performance is enhanced for both strength and speed. Such a situation would yield a negative correlation at the among‐individual level (trade‐off), but a positive correlation at the within‐individual level (training effect), in which case multivariate mixed models should be used to partition the relationships at the two levels. Otherwise, the trade‐off occurring at the among‐individual level can be masked by within‐individual changes in condition).

Performance traits provide some of the best examples of functional trade‐offs (i.e. when features that enhance performance of one task decrease performance of another; Garland, Downs & Ives, 2022). Indeed, several performance trade‐offs are relatively well understood on the basis of the biochemical and morphological properties of organisms (Castro *et al*., 2024; Gvož & Van Damme, 2006; Husak & Lailvaux, 2022; McHenry & Summers, 2011; Wilson & James, 2004). However, performance trade‐offs can be relatively hard to detect within populations unless a proper (co)variance partitioning approach is adopted (Berberi & Careau, 2019; Careau & Wilson, 2017a,b; Lailvaux, Cespedes & Houslay, 2019; Lang & Gifford, 2023). For example, one may intuitively expect that performance trade‐offs are driven by among‐individual variation in phenotypes (e.g. morphological and physiological traits) that enhance certain types of performance while impairing others, but positive within‐individual correlations can occur because of unquantified variation in body condition (e.g. fat content, hormonal state) or extrinsic factors (e.g. diet, training). This situation has been coined the ‘sink or swim’ scenario (Careau & Wilson, 2017a), because it was first illustrated in a simulated population of Krakens that face a trade‐off between swimming and ship‐sinking performance (Fig. 2). Two studies illustrate such a scenario: one on decathletes and heptathletes (where trade‐offs among throwing, jumping, and running performance occurred at the among‐individual level while positive within‐individual correlations occurred as a result of unquantified variation in some factors affecting performance on all events similarly; Careau & Wilson, 2017b); and one on wild white‐footed mice (*Peromyscus leucopus*) (where a trade‐off between grip strength and sprint speed occurred at the among‐individual level, but the two traits tended to be positively correlated within individuals; Berberi & Careau, 2019).

Performance traits are linked to numerous lower‐level traits (Foster *et al*., 2015), including at the levels of physiology (e.g. muscle fibre type composition, Ca^2+^ cycling), morphology (Bennett, 1989; Garland & Losos, 1994), the endocrine system (Husak *et al*., 2009), and metabolism in general (Careau & Garland, 2012). Arnold's (1983) idea that structural equation modelling or path analysis can be used to analyse such relationships simultaneously for multiple measures of performance, as well as the pathways from performance measures to components of Darwinian fitness, has since become an intellectual cornerstone of evolutionary physiology and related fields (e.g. Addis *et al*., 2017; Cooke *et al*., 2014; Dantzer, Westrick & van Kesteren, 2016; Garland & Losos, 1994; Higham *et al*., 2021; Husak & Lailvaux, 2017; John‐Alder *et al*., 2009; Khan *et al*., 2024; Storz *et al*., 2015). Nonetheless, due largely to the difficulty of measuring traits at multiple levels of biological organisation for the same set of individuals (see Section IV.3), empirical studies that use path analysis to integrate across multiple levels in the morphology–performance–behaviour–fitness hierarchy are rare (Albuquerque, Zani & Garland, 2023; Matthews *et al*., 2023) and other analytical approaches have been used (McCormick, Fakan & Allan, 2018).

### (2) Behaviour and the rise of quantitative genetics in behavioural ecology

Although the definition of behaviour (Calhoun & El Hady, 2023; Levitis, Lidicker & Freund, 2009) is, if anything, more complicated than the definition of performance, for our purposes it can be taken to mean anything that an animal does or fails to do (see also Table 1). As a field focusing on the adaptive nature of behaviour, behavioural ecology has experienced many shifts in the topics of interest and types of questions addressed. In the 1960s, foraging, fighting, sex, and cooperation were the main research foci (Krebs & Davies, 1984). The field experienced huge growth in the 1970s and 1980s, with research topics like optimal foraging and mating systems, which then shifted to sexual selection and host–parasite interactions in the 1990s to early 2000s (Owens, 2006). These research foci set a very fertile ground for studying the adaptive nature of animal behaviour, yet the first ~50 years of behavioural ecology research have largely ignored quantitative genetics (but see Boake, 1994). Instead, behavioural ecologists took the ‘phenotypic gambit’ (i.e. make the assumption that phenotypic patterns of (co)variation are reflective of the underlying genetics; Grafen, 1984).

**Table 1:** Definitions of key concepts and phenotypic traits.

Term (abbreviation)	Definition
Behaviour	The internally coordinated responses (actions or inactions) of whole living organisms to internal and/or external stimuli, excluding responses more easily understood as developmental changes (Levitis *et al*., 2009).
Behaviour–performance continuum	A conceptual axis along which can be placed various behavioural and performance traits, depending on the relative influence of behavioural choice and internal *versus* external motivation. See Fig. 1.
Coadaptation	Occurs when two or more traits interact with each other to affect fitness, that is, they are under correlational selection (Sinervo & Svensson, 2002). Note that the term coadaptation is different than coevolution, which is the evolution in two or more species in which the evolutionary changes of each species influence the evolution of the other species.
Fitness (Darwinian)	The lifetime reproductive success (i.e. number of viable descendants) of an individual.
Fitness (physical)	The ability of the body to perform various aspects of daily activities, occupations or sports.
Individual variation	The phenotypic diversity within a population after accounting for age and sex differences.
Lower‐level traits	Typically, morphological (e.g. leg length) or physiological (e.g. blood pressure) traits that are not directly linked to Darwinian fitness. Also referred to as subordinate traits.
Motivation	An internal state of mind that compels individuals to engage in behaviour. Motivation is affected by both internal (e.g. hunger) and external (e.g. presence of a predator) factors. Multiple measures can be used to assess its level.
Performance (maximal, laboratory)	The ability of an individual to conduct a task when maximally motivated. Arnold (1983) specified that a performance trait should preferentially be ecologically relevant and comparable across species. Husak *et al*. (2009) recognised two primary categories: dynamic performance that includes measurements of movements of the whole body, or parts of the body (e.g. sprint speed, endurance, and bite force), and regulatory performance that includes measures of how well organisms regulate physiological processes of the whole body, or withstand environmental conditions (e.g. regulation of salt and water, thermoregulation or thermal tolerance, growth, digestive capacity, and immune response).
Performance (ecological)	How an organism performs in nature while accomplishing an ecologically relevant task (Irschick, 2003; Irschick & Garland, 2001).
Performance test	A series of multiple performance trials conducted on a given individual within a short time period (typically a day).
Performance trial	A recorded output (e.g. speed, force) on given individual as it is prompted to perform a given task.
Personality (broad sense)	Repeatable individual differences in behaviour (Réale *et al*., 2010).
Personality (narrow sense)	Repeatable individual difference in activity, exploration, boldness, aggressiveness, and sociability (Réale *et al*., 2010).
Personality trait – activity	The general level of physical activity of an individual, in terms of muscular movement leading to locomotion (Réale *et al*., 2007).
Personality trait – aggressiveness	An individual's agonistic reaction towards conspecifics (Réale *et al*., 2007).
Personality trait – boldness	An individual's reaction to a risky but non‐novel situation. Docility, tameness, and fearfulness have been used in the specific context of reaction to humans (Réale *et al*., 2007).
Personality trait – exploration	An individual's reaction to a novel situation, including behaviour towards a novel environment, habitat, food item, or object. A novel situation can also be considered risky if, for example, a new object may represent a potential predator (Réale *et al*., 2007).
Repeatability (τ, *R*)	The proportion of total phenotypic variance that is due to consistent differences among individuals (Falconer & Mackay, 1996), usually measured as the intra‐class correlation coefficient (Lessells & Boag, 1987), Pearson product–moment correlation (Hayes & Jenkins, 1997), or individual identity as a random effect in a mixed model (Wilson *et al*., 2010).
Standardised test	A protocol used to measure performance and/or behaviour under a certain defined set of conditions, in the hope to control for confounding variables that could generate unwanted variation among and within individuals within a study (or among studies).
Trade‐off	Most simply, when one trait cannot increase without a decrease in another (Garland, 2014), although not all types of biological trade‐offs are this simple (Garland *et al*., 2022).

In the early 2000s, a revolution in the field of behavioural ecology arose from the integration of concepts and techniques from the field of quantitative genetics (Réale *et al*., 2007; Sih, Bell & Johnson, 2004a). The resulting focus on repeatable individual differences in behaviour across contexts or time led to the rapid emergence of ‘animal personality’ as a topic of major interest. Although the study of personality in non‐human animals and use of the term ‘animal personality’ was not entirely original (Arnold & Bennett, 1984; Budaev, 1997; Draper, 1995; Herzog & Burghardt, 1988; Yerkes, 1939), a change of focus to individual differences certainly represents an important step for behavioural ecology, because the highly labile nature of behavioural traits makes it perilous to assume that a phenotypic correlation reflects the among‐individual correlation (Bell, Hankison & Laskowski, 2009; Brommer, 2013). Moreover, the (co)variance partitioning approach needed to quantify repeatability and among‐individual correlations thoroughly (Dingemanse & Dochtermann, 2013) naturally lends itself to the more complex (and data‐hungry) models needed to quantify key microevolutionary parameters [e.g. heritability and genetic correlations (Wilson *et al*., 2010); see Section IV.4]. Overall, additive genetic variance accounts for about half (52%) of the animal personality variation (Dochtermann, Schwab & Sih, 2015), which implies the presence of other important sources of repeatable individual differences in behaviour, such as individual (unique) experience, maternal effects, and epigenetic mechanisms (Laskowski *et al*., 2022; Sepers, Verhoeven & van Oers, 2024).

Besides repeated measures and (co)variance partitioning (Dingemanse & Wright, 2020), a main feature of animal personality studies is the use of somewhat standardised tests (Careau & Garland, 2012). A wide range of standardised tests are used to capture aspects of activity, exploration, boldness, aggressivity, and sociability. Exactly what a given test measures depends on the degree of novelty, social context, and perceived risk to the animal, among other factors. [Note that although the tests are standardised across individuals within a study, details often vary from study to study, even within the same laboratory, e.g. for different versions of the open‐field test, see Bronikowski *et al*. (2001) and Careau *et al*. (2012)]. The appeal of using standardised tests stems from the fact that behaviour is extraordinarily sensitive to small differences in the testing conditions (Crabbe, Wahlsten & Dudek, 1999), and so is not as reliable or reproducible as, say, morphology, with measures of physiology and performance most likely somewhere in between (Boake, 1989; Nespolo & Franco, 2007).

### (3) The behaviour–performance continuum

The various tests used to measure performance and behaviour capture traits that are influenced by varying levels of stress and motivation. These traits can be positioned along a behaviour–performance continuum that ranges from what animals voluntarily *do* when given the choice (i.e. behaviour) to what they *can do* when maximally motivated (i.e. performance; Fig. 1). In addition to motivation, an individual's reaction to any testing situation that is novel or perceived as risky will be greatly influenced by its physiological and psychological responses, which have been termed its ‘stress coping style’ (Koolhaas *et al*., 1999; Perals *et al*., 2017). Although some tests clearly capture performance [e.g. endurance on a treadmill with electrical stimulation as motivation and some attempt at physiological validation of exhaustion; see Booth, Laye & Spangenburg (2010) and Meek *et al*. (2009)] *versus* voluntary behaviour (e.g. voluntary wheel running; Abdeen, Agnani & Careau, 2022), many other tests are harder to categorise. This is especially the case for performance tests that rely primarily on the animal's intrinsic motivation [e.g. bite force (see Section III.5), or maximum size of a prey item swallowed], rather than an external stimulus. Also difficult to categorise are studies that, for example, tie the delivery of food to the performance of a task, such as running in a wheel (Fonseca *et al*., 2014; Vaanholt *et al*., 2007).

Ultimately, the position along the behaviour–performance continuum has to do with the experimental protocol. When measuring performance, study organisms are usually subjected to a test in which they have very limited choice other than performing the required task, and are ‘motivated’ to do so in various external ways (mild electric shocks, chasing, tapping of the tail, etc., e.g. Albuquerque, Bonine & Garland, 2015; Layne & Benton, 1954; Lerman *et al*., 2002). If, on the other hand, the goal is to measure behaviour, then animals can be directly observed in their natural (or artificial) habitat, without disturbance (e.g. Albuquerque *et al*., 2023; Garland, 1999; Perry *et al*., 2004), or they might also be approached by the investigator or presented with a specific novel or risky situation (e.g. introduction of predator cues) to which their reactions are recorded (e.g. distance moved, latency to leave a refuge). In behavioural observations or behavioural tests, animals are usually left with a wider range of potential actions they can take, and aside from setting up the initial conditions, the observer has a very limited role. This is the opposite of performance tests, where the observer actively tries to ensure the execution of the desired task by the animal (e.g. running in a straight line at maximum speed).

Some specific tests measure output traits that are differently labelled as performance or behaviour, depending on the context. Sprint speed, for example, when measured by chasing an individual along a racetrack in the laboratory, or by chasing it upon release back into its natural habitat, is often considered a measure of locomotor performance (Djawdan & Garland, 1988; Irschick *et al*., 2008). One could also argue that running away from a threat (in this case a human) is a measure of escape behaviour, in a context of fight or flight response (Evans *et al*., 2019). In both cases, researchers are measuring the same thing: the speed at which the individual runs to escape. In practice, however, the interest of the researchers may define which label is used, which creates confusion and raises important questions. At which point is a running speed measurement likely to yield a valid measure of sprint performance ability *versus* ‘only’ escape behaviour? Simply releasing an animal – without chasing it – may be unlikely to measure its maximal running speed. Accordingly, Piquet *et al*. (2018) used escape trials to assess running speed in Barbary ground squirrels (*Atlantoxerus getulus*), but they did not chase or otherwise stimulate the squirrels to attain their maximum running speed and therefore considered that their measure better reflected behaviour rather than performance.

If additional stimulation is needed for measurements to qualify as performance, then what kind of stimulation and how much? Should the animal simply be encouraged to run by shouting and arm‐waving upon release (Blumstein, 1992) or does it need to be chased (e.g. Djawdan & Garland, 1988)? Or, is it necessary to reach a point where animals show signs of distress or possibly even injuries to consider the measurement to have achieved maximal performance ability? These questions raise important conceptual, practical, and ethical considerations. We think the behaviour–performance continuum (Fig. 1) can provide guidance, as it can help researchers position the traits they measure along a continuum instead of forcing them to label them in a dichotomous manner as ‘behaviour’ *versus* ‘performance’. For example, the measurements of escape speed in Piquet *et al*. (2018) may well constitute a behavioural trait, but these measurements lie closer to the performance end of the continuum than the other behaviour measured in Piquet *et al*. (2018) (i.e. latency to enter a novel environment).

### (4) Ecological performance

Considering the behaviour–performance continuum becomes even more important given the various contexts under which locomotor performance is measured. As presented by Arnold (1983), performance is usually measured in the laboratory and assumed or intended to represent the *ability* of animals to conduct ecologically relevant tasks in their natural habitat (Irschick & Garland, 2001). However, free‐ranging animals often only use a portion of their locomotor abilities, and this proportion might vary depending on other factors, such as sex and age (Irschick, 2003). Perhaps in an attempt to stress the importance of the context within which performance is measured, the term ‘ecological performance’ was coined to represent how organisms utilise maximal performance in nature in various contexts (Husak, 2006b; Irschick, 2003; Irschick & Garland, 2001; Yap, Serota & Williams, 2017). Yet, if discussing ecological performance allows better differentiation between measurements of performance made in the field *versus* laboratory, then the distinction between performance and behaviour starts to blur as a result (see Table 1 for definitions).

As explained above, performance is typically measured in the laboratory, or in the wild with a certain degree of human intervention, to generate external motivation and limit behaviour choice. By contrast, ecological performance is measured on a free‐ranging animal as it competes with conspecifics, chases prey, and escapes predators. Given the highly heterogenous nature of natural habitats, myriad (un)controlled factors can generate variation in ecological performance measurement; for example, the speed at which an animal escapes from a human threat increases with distance from a refuge (Braña, 2003; see also Bulova,^1994^). As such, ecological performance traits may fall closer to the behavioural end of the continuum. For example, a common way to measure ecological performance involves walking towards an animal at a constant speed and recording the speed at which it flees (Husak & Fox, 2006; Irschick & Losos, 1998). The same protocol is frequently used to measure ‘boldness’ in free‐ranging animals, except that in this case the observer measures the distance at which the animal initiated the fleeing response (Blumstein, 2003; Møller, 2009). In reality, the distance and speed at which an animal flees from a putative predator are both important aspects of the anti‐predator response (e.g. see fig. 11.1 in Foster *et al*., 2015) that are likely co‐adapting in an additive or compensatory way (Cooper, Pyron & Garland, 2014) (see the co‐specialisation and compensation hypotheses in Section IV.1).

It is intuitive to think of ecological performance as being below maximal abilities, but Jayne & Ellis (1998, p. 1128) found that ‘the average maximal velocity (2.79 m/s) observed for the level laboratory trials was either 77 or 71% of estimated field values, depending on the method used.’ One interpretation would be that the lizards studied by Jayne & Ellis (1998) could not be maximally motivated in the laboratory. Indeed, some species (e.g. kangaroo rats (*Dipodomys* spp.) in Djawdan & Garland, 1988) will not perform at maximum in the laboratory, which means that, by definition, ‘performance’ cannot be measured in laboratory trials for those species and performance measures taken in the field may yield a better indicator of maximum abilities. An alternative interpretation is that studies comparing individual ecological performance values to species average maximal values are inconclusive. Indeed, some studies have attempted to quantify the percentage of maximal performance used by wild animals, which led to the proposal of so‐called ‘overachievers’ and ‘slackers’ (Irschick *et al*., 2005, p. 1582). However, this approach can lead to spurious results if based on different samples of individuals measured in the laboratory *versus* wild. To understand how much of maximal abilities are used, it is important to measure maximal and ecological performance on the same individuals, for example as done by Husak & Fox (2006), who showed that faster lizards used a lower percentage of their maximal capacity (as measured in the laboratory) when foraging and escaping predators in the field.

Clearly, ecological performance is a complicated topic, and variation in the protocols used across studies implies that performance includes a mix of measurements made in the field and in the laboratory, on animals that are prompted to perform by various techniques that range from a simple approach towards an animal until it escapes (e.g. Jayne & Ellis, 1998) to clearly frightened animals chased by a putative predator. This undoubtedly generates great variation in measures of locomotor performance, and it is important to recognise that some of that variation has to do with positioning relative to the performance end of the continuum (Fig. 1).

### (5) Variation in motivation among and within individuals

From a practical standpoint, measuring such traits as maximum jumping distance, maximum speed during flight, and running endurance requires equipment with high precision and accuracy. Complications quickly arise because not all individuals and/or species will perform the required task when prompted to do so. These complications are particularly challenging for such traits as bite force (e.g. Herrel *et al*., 2005) and flight speed (e.g. Chai *et al*., 1999). To measure bite force, an animal must bite on a force transducer, but will typically refuse to perform if not intrinsically motivated or aggressive enough (Freeman & Lemen, 2008). Similarly, to measure flight speed, an animal must fly in an apparatus like a wind tunnel, but some will refuse. In these cases, differentiating between performance and behaviour is difficult, because they presumably both contribute to the measured output recorded as bite force or flight speed. Related to these issues, studies in rodent exercise physiology often train animals to run on a treadmill for a couple of days before implementing the actual performance trials, and individuals that do not cooperate or perform well may be excluded from the study (e.g. Koch *et al*., 1998; Lutton & Hudson, 1980).

None of the considerations above are new: Losos *et al*. (2002, p. 58) noted that motivation was a major problem ‘bedevilling studies of performance’ and highlighted the dangers of discussing performance measurements when animals can vary in their propensity (motivation) to perform. As noted at the start of this review, a common way of tackling this problem is to use ‘personal best’ values (i.e. selecting the trial with the highest output to eliminate sub‐maximal trials). Although this greatly reduces or even eliminates within‐individual variation in motivation, the possibility will always remain that individuals differ from one another in their overall motivation when prompted to perform (Fiedler & Careau, 2021). In this case, two individuals with different intrinsic performance capacities may nevertheless yield the same measured output, if the individual with the higher intrinsic performance is less motivated than the other – yet maximally motivated – individual.

In any case, selecting maximal values out of repeated measurements introduces problems (Adolph & Hardin, 2007; Adolph & Pickering, 2008). Because of its nature, the sample distribution of extreme values introduces bias (Head, Hardin & Adolph, 2012), and when the sampling is unequal among individuals, even greater bias emerges because more frequently sampled individuals are more likely to reach higher values. Instead of using personal best values, one approach is to retain all of the ‘good’ repeated measurements made on every individual and use multi‐level statistical tools to partition variance accordingly (Careau & Wilson, 2017a). By ‘good’ measurements, we mean any trial that was not obviously submaximal based on visual observations during testing (e.g. Jayne & Ellis, 1998; Losos *et al*., 2002). Applying a variance partitioning approach to such data allows among‐individual variation to be quantified separately from within‐ or intra‐individual variation (i.e. how performance differs across individuals *versus* how the performance of an individual varies over time or from one test to another; see Section IV.2). Presumably, motivation also generates variation at the among‐ and within‐individual levels, but evaluating this possibility remains a challenging task because it is almost impossible to quantify motivation separately from the performance measurements.

One useful approach is to develop an indicator of the willingness or cooperativeness of the animal (e.g. Chappell *et al*., 2007; Claghorn *et al*., 2017; Coleman *et al*., 1998; Le Galliard *et al*., 2013; Rezende *et al*., 2005; Swallow *et al*., 1998). These indicators can be subjective (e.g. did the animal run consistently or inconsistently) or rely on simple metrics with unknown physiological effects (e.g. the number of shocks or air puffs or taps as a measure of how much stimulation an animal needs to be motivated) (e.g. Kregel *et al*., 2006; Le Galliard *et al*., 2013; Lutton & Hudson, 1980; Sorci *et al*., 1995). For example, Swallow *et al*. (1998) used a subjective assessment of run quality (five categories from poor to excellent) in mice running on a treadmill with both manual and electrical stimulation. This indicator was statistically repeatable between two daily trials and was positively affected by 8 weeks of access to running wheels. Trial quality was also used as a covariate in analyses of *V*O_2max_, where it was not a significant predictor of *V*O_2max_, and of the maximum speed attained during the VO_2max_ trials, where it was a significant positive predictor.

Returning to lizards, Sorci *et al*., (1995) recorded the number of manual stimuli (range = 0–13) required when chasing lizards along a racetrack during speed trials, and it was a positive predictor of sprint speed. Interestingly, calculated heritabilities were higher for raw than for stimuli‐corrected sprint speed. They also argued that stimuli‐corrected measures of speed should correspond more to a ‘physiological’ measure of speed, i.e. maximum performance ability. Further, they suggested that natural selection likely acts on ‘realised’ speed, which is the result of the interaction between the animal's physiological capacity and its motivational state, and so uncorrected measures of speed might be more ‘ecologically relevant’.

According to Booth *et al*. (2010, p. 220), however, ‘no studies have proven that the frequency that a rodent contacts an electric shock is associated with any of the other aforementioned measurements of exhaustion.’ Booth *et al*. (2010) also recognised that a potential problem with employing a grading scale solely based on number of contacts to electric shocks is that individuals typically exhibit various ‘running styles with some styles causing premature stopping before biochemical exhaustion’ (Booth *et al*., 2010, p. 220). In Copp *et al*. (2009), for example, some rats likely fatigued earlier than others because of the excess energy they spend fighting the treadmill belt for an extended time before demonstrating a natural running gait. For that reason, Copp *et al*. (2009) recommend excluding these animals from exhaustion tests. By excluding individuals based on their (lack of) motivation to perform under forced exercise, we may introduce a bias related to aspects of stress coping styles and personality. How much of a problem can this be?

An important step at this point is to determine the importance of among‐ *versus* within‐individual differences in motivation during forced‐exercise tests: if the individual repeatability (*R*) of a measure of motivation is close to 0, then exclusions based on motivation would be less problematic than if *R* is close to 1. We are aware of only two studies that have quantified *R* in the motivation to perform under forced exercise. Swallow *et al*. (1998) obtained *R* = 0.49 for run quality, which implies that some individuals were consistently less motivated than others to perform, and these low‐motivation individuals would be more likely to be excluded from performance studies. Fiedler & Careau (2021) conducted repeated forced‐exercise tests on a large number of wild mice (*Peromyscus leucopus*) and found that the repeatability of willingness to run on the treadmill was *R* = 0.16 ± 0.08. Although that repeatability estimate is reassuringly low, Fiedler & Careau (2021) had to exclude ~10% of the individuals tested because their unwillingness to run resulted in obviously sub‐maximal measures. Perhaps the exclusions were acceptable in the context of their study, but what if the objective was to test for a relationship between performance and behavioural traits? For example, Le Galliard *et al*. (2013) found weak phenotypic correlations (*r* < 0.16) between motivation to perform and behaviour measured in a novel environment, suggesting that excluding the least‐motivated individuals from the study should not introduce a ‘personality‐related bias’ [note that Le Galliard *et al*. (2013) did not evaluate *R* in their quantitative measures of motivation and did not specify that any individuals were excluded from their study due to lack of motivation during performance measurements].

If we want to understand how performance and behavioural traits evolve in a coordinated fashion, we must know the extent to which correlations are driven by causal mechanisms (e.g. pleiotropic gene action acting through effects on the endocrine system) relative to methodological issues and potential measurement bias. But what does the current literature have to say about the correlation between performance and behavioural traits?

### (6) The need for a literature review on behaviour and performance

Are performance and behavioural traits correlated, and, if so, how? In trying to answer this question, a significant hurdle presents itself (in addition to the conceptual and technical challenges identified above): inconsistent terminology. Although we do not lack studies on locomotor performance itself, any attempt at synthesising the field will face the problem that scientists from a wide array of disparate fields use the term ‘performance’ to designate various traits deemed desirable in some way or another. This tendency became clear during our literature search (see Section II), where a large number of studies contained the term ‘performance’, but only a very small fraction of these were referring to the actions of an organism that is presumably maximally motivated to accomplish a task.

Scientists strive towards clear and meaningful definitions because failure to do so may hinder progress [see de Queiroz & Donoghue (1988) and Levitis *et al*. (2009) for examples], but the range of meanings of ‘performance’ is staggering. Apart from its main definition as the ability of an individual to perform a task when maximally motivated (Careau & Garland, 2012; Garland & Losos, 1994), performance is sometimes used to refer to reproductive performance (e.g. Das *et al*., 2016; Festing, 1968), including mating success (e.g. Byers, Hebets & Podos, 2010) or reproductive success (e.g. Lane & Briffa, 2022). However, using performance in this context relies on the (implicit) assumption that individuals are always maximally motivated to mate and produce as many offspring as possible, which may not always be true (e.g. in many cases a greater lifetime reproductive success may involve producing fewer offspring than maximally possible in a given reproduction event). Performance is also used to refer to how well an individual behaves in its natural habitat in comparison to other individuals, for example courtship displays (e.g. Manica *et al*., 2017) or fighting (e.g. Edmonds & Briffa, 2016). Performance also sometimes refers to regulatory performance (Husak *et al*., 2009), more specifically, to the ability of animals to regulate physiological processes, such as growth rate, thermoregulation, or even digestive capacity and production of gametes (Coleman & Moore, 2003; Sandberg, Emmans & Kyriazakis, 2007; Seebacher, 2005). In the agricultural literature, performance often refers to traits of economic importance, such as milk yield (Baumgard, Collier & Bauman, 2017; for mice, see also Josefson, De Moura Pereira & Skibiel, 2023). In some cases, traits that are clearly behavioural are referred to as performance (e.g. Whimbey & Denenberg, 1967). Because of this wide range of definitions, searching for studies on the relationship between locomotor performance and behaviour can be challenging.

Here, we conducted a literature review of how locomotor performance and behaviour covary in animals. We believe that such information will be useful to guide future research on locomotor performance and its relationship with other ecologically relevant traits. We provide a review of published research articles reporting any relationship between locomotor performance and behavioural traits. In so doing, we realised that a growing list of studies reports links between performance and behaviour across many taxa, that the vast majority of those studies report phenotypic correlations, and that only a handful have explored potential correlated responses to selection on performance or behaviour. We discuss our findings in relation to two hypotheses that seek to explain the relationship between behaviour and performance traits, and outline a few recommendations to help guide future studies.

## II. LITERATURE SEARCH

We conducted a literature survey of existing primary studies to assess relationships between locomotor performance and behavioural traits in both field and laboratory settings. We only included studies that tested for links between locomotor performance and behavioural traits that qualify under our definitions (see Table 1). Although the categorisation of traits as behaviour *versus* performance might go against the proposed continuum, we made an effort to label traits as behaviour when the animals were given the choice of what to do and performance when there was an external source of motivation to conduct a specific task (and generally limit behavioural options). Typically, behavioural and performance traits are quantified using separate assays, but we included studies in which behaviour was quantified during a ‘free will’ portion just before performance was measured using forced exercise. For example, Kasumovic & Seebacher (2018) conducted trials in which golden orb‐web spider (*Nephila plumipes*) were allowed to climb 40 cm on a strip of masking tape while undisturbed to determine casual (voluntary) locomotor speed, after which maximal locomotor speed was measured by chasing the spider with a soft paintbrush for an additional 40 cm. However, and contrary to a recent meta‐analysis (Wu & Seebacher, 2022), we excluded studies that extracted aspects of ‘performance’ during a behavioural test. For example, the fastest running speed voluntarily displayed by a rodent on a running wheel does not correspond to performance according to our definition (i.e. it is behaviour), and for that reason the studies by Rezende *et al*. (2009) and Chappell *et al*. (2004) were not included. Similarly, we excluded studies that only extracted aspects of behaviour during a performance test [e.g. the average jumping distance during a series of forced jumps (Rogowitz & Sánchez‐Rivoleda, 1999; Walton, 1988)]. Another example is the number of pauses in the running sequence and longest distance covered between pauses during burst speed trials on a racetrack (Braña, 2003).

The original literature search was conducted on July 8 2022 on the *Web of Science* website in the ‘topic’ field, including all available years. We used a list of key words as follows: ‘performance, sprint speed, sprint, escape, endurance, swimming, running, jumping, jump, run’ AND ‘behavior, behaviour, exploration, shyness, boldness, activity, personality, temperament’. Our search string used the ‘AND’ argument to return studies only that included key words in both categories (performance and behaviour). Once non‐relevant areas of research were excluded, the original search returned a total of 22,531 studies.

We then proceeded in two steps; first, we screened abstracts to pre‐select studies and then read all pre‐selected studies to extract information. In the first step, abstracts were ordered by relevance according to *Web of Science*, and as we progressed and relevance decreased, fewer and fewer studies were pre‐selected. After no studies could be pre‐selected (between studies ranked 8,000–9,000), we only screened 100 abstracts out of every 500, which did not result in any more studies being retained. A total of 337 studies were pre‐selected in this first step and saved as a ‘first scan’ list. In a second step, we read every pre‐selected study to assess suitability to extract information, which resulted in a total of 57 research articles containing tests for the links between locomotor performance and behaviour. Some studies published after our search were also included as we became aware of them (Agnani & Careau, 2023; Albuquerque *et al*., 2023; Courtene‐Jones & Briffa, 2023; Khan *et al*., 2024; and unpublished data from N. Bonin & Careau, in preparation). We note that our methods do not follow PRISMA guidelines for systematic reviews and meta‐analyses (Moher *et al*., 2015). Therefore, care should be taken when interpreting the results as they might not represent the literature in general.

Studies were grouped into three categories. First, the most common type of study quantified one or more correlations between locomotor performance and behavioural traits measured on a common set of individuals. These 45 studies are reported in Table 2 along with their effect sizes. Second, eight studies quantified how performance and behaviour changed in response to a manipulation of the environment (e.g. temperature, substrate type: Table 3). These studies are informative for the link between performance and behaviour because they provide evidence for correlated plasticity (i.e. when two or more traits change in response to a common environmental variable). Third, we compiled four evolution experiments in which artificial selection (Garland & Rose, 2009) was applied on performance and resulted in a correlated change in behaviour, or *vice versa* (Table 4). Only one relevant study was excluded from Table 4 because it reported no significant correlated changes in performance traits in response to selection on behaviour (Khan *et al*., 2024).

**Table 2:** List of included articles reporting associations between measures of locomotor performance and behavioural traits. When more than two traits were significantly correlated, each relationship appears as a different row. Descriptions of performance measurements and behaviour are shortened but close to the original descriptions. OF = open field. Effect sizes are reported as correlations (*r*_P_ = phenotypic correlation; *r*_ind_ = among‐individual correlation; *r*_*e*_ = within‐individual correlation; *r*_env_ = environmental correlation; *r*_G_ = genetic correlation; *r*_P‐ind_ = correlation between the repeatable part of a repeatedly measured trait and the phenotypic variation in a singly measured trait); *N*_ID_ = number of individuals sampled; *n*_obs_ = number of observations; Prob. = probability; Dur. = duration, Nbr. = number; Avg. = average; Max = maximum; Dist. = distance. When needed, estimates were multiplied by −1, such that a positive correlation indicates higher performance is associated with higher levels of locomotor activity (movement) exploration, and boldness. Statistically significant correlation estimates are indicated in bold. If the correlation was not available, the relationship is indicated as significant (SIG) or not significant (NS).

Taxa		Performance			Behaviour			Level	*r* ± se			Ref.
		Trait	*N*_ID_	*n*_obs_	Trait	*N*_ID_	*n*_obs_					
Arthropods												
	*Lycaena tityrus*	Endurance	301	301	Exploration	301	301	*r*_P_	**0.19 ± 0.06**			Reim *et al*. (2019)
		Endurance	165	165	Exploration	165	165	*r*_P_	**0.22 ± 0.07**			
	*Hogna carolinensis*	Sprint speed	25	25	Flight initiation distance (× − 1)	25	25	^1^ *r*_P_	**−0.49 ± 0.16**			Nelson & Formanowicz (2005)
	*Nephila plumipes*	Max climbing speed	57	57	Casual climbing speed	57	57	*r*_P_	**0.46 ± 0.11**			Kasumovic & Seebacher (2018)
		Max climbing speed	33	66	Casual climbing speed	33	66	^3^ *r*_ind_	**0.65 ± 0.25**			
	*Pagurus bernhardus*	Sprint speed	51	51	Startle dur. (× − 1)	51	407	^3^ *r*_P‐ind_	**−0.24 ± 0.14**			Courtene‐Jones & Briffa (2023)
	*Teleogryllus commodus*	Jump distance	817	817	Calling effort	924	924	^1^ *r*_G_	−0.04			Lailvaux *et al*. (2010)
		Jump power	817	817	Calling effort	924	924	^1^ *r*_G_	−0.13			
Fish												
	*Danio rerio*	Critical swimming speed	24	24	OF speed	24	24	*r*_P_	**0.71 ± 0.11**			Seebacher *et al*. (2015)
		Critical swimming speed	42	42	OF speed 1st min	42	42	*r*_P_	**0.66**			Seebacher *et al*. (2016)
		Critical swimming speed	42	42	OF speed 10th min	42	42	*r*_P_	NS			
	*Cyprinodon pecosensis*	Critical swimming speed	46	46	Territoriality	46	46	*r*_P_	NS			Kodric‐Brown & Nicoletto (1993)
	*Oncorhynchus mykiss*	Swimming endurance	12	12	Nbr upstream transits	12	12	*r*_P_	**0.93**			McDonald *et al*. (2007)
	*Salvelinus fontinalis*	Endurance	73	146	Time spent moving	73	73	*r*_P_	0.01			Farwell & McLaughlin (2009)
	*Poecilia reticulata*	Maximal swimming speed			Dispersal				NS			Le Roy & Seebacher (2018)
	*Pomacentrus chrysurus*	Maximum escape speed	110	110	Thigmotaxis (outer zone) (× − 1)	110	110	*r*_P_	0.10 ± 0.09			McCormick *et al*. (2018)
		Maximum escape speed	111	111	Lateralisation (absolute)	111	111	*r*_P_	−0.10 ± 0.09			
		Maximum escape speed	111	111	Lateralisation (relative)	111	111	*r*_P_	−0.17 ± 0.09			
		Maximum escape speed	110	110	Routine distance moved	110	110	*r*_P_	0.00 ± 0.10			
		Maximum escape speed	111	111	Bite rate	111	111	*r*_P_	0.17 ± 0.09			
		Maximum escape speed	111	111	Total distance moved	111	111	*r*_P_	0.08 ± 0.09			
		Maximum escape speed	111	111	Boldness	111	111	*r*_P_	0.14 ± 0.09			
Amphibians												
	*Anaxyrus boreas*	Jumping distance	60	60	Distance moved	60	60	^2^ *r*_P_	**0.22 ± 0.12**			Bredeweg *et al*. (2019)
	*Hyliola regilla*	Jumping distance	60	60	Distance moved	60	60	^2^ *r*_P_	**0.34 ± 0.11**			
	*Rana cascadae*	Jumping distance	60	60	Distance moved	60	60	^2^ *r*_P_	0.09 ± 0.13			
	*Xenopus tropicalis*	Maximum swimming speed	86	389	Total distance moved	84	252	^4^ *r*_ind_	**−0.69 ± 0.19**			Videlier *et al*. (2014)
		Maximum swimming speed	86	389	Mean movement speed	84	252	^4^ *r*_ind_	−0.28 ± 0.19			
		Maximum swimming speed	86	389	First movement latency (× − 1)	84	252	^4^ *r*_ind_	**0.65 ± 0.27**			
		Maximum swimming speed	86	389	Time spent moving	84	252	^4^ *r*_ind_	**−0.84 ± 0.25**			
		Maximum swimming speed	86	389	Nbr movements away from wall	84	252	^4^ *r*_ind_	**−0.45 ± 0.20**			
		Endurance (distance)	84	480	Total distance moved	84	252	^4^ *r*_ind_	0.10 ± 0.22			
		Endurance (distance)	84	480	Mean movement speed	84	252	^4^ *r*_ind_	−0.22 ± 0.19			
		Endurance (distance)	84	480	First movement latency (× − 1)	84	252	^4^ *r*_ind_	0.09 ± 0.27			
		Endurance (distance)	84	480	Time spent moving	84	252	^4^ *r*_ind_	0.00 ± 0.27			
		Endurance (distance)	84	480	Nbr movements away from wall	84	252	^4^ *r*_ind_	0.07 ± 0.21			
		Endurance (time)	84	480	Total distance moved	84	252	^4^ *r*_ind_	**0.48 ± 0.18**			
		Endurance (time)	84	480	Mean movement speed	84	252	^4^ *r*_ind_	−0.09 ± 0.17			
		Endurance (time)	84	480	First movement latency (× − 1)	84	252	^4^ *r*_ind_	−0.24 ± 0.25			
		Endurance (time)	84	480	Time spent moving	84	252	^4^ *r*_ind_	0.34 ± 0.24			
		Endurance (time)	84	480	Nbr movements away from wall	84	252	^4^ *r*_ind_	0.34 ± 0.18			
	*Bufo calamita* (CE environment)^6^	Sprint speed	118	118	OF latency (× − 1)	118	118	*r*_P_	0.14			Maes *et al*. (2012)
		Sprint speed	118	118	OF activity	118	118	*r*_P_	0.19			
		Sprint speed	118	118	OF max speed	118	118	*r*_P_	0.18			
		Sprint speed	118	118	OF avg speed	118	118	*r*_P_	0.10			
		Sprint speed	118	118	OF exploration	118	118	*r*_P_	0.06			
		Endurance	118	118	OF latency (× − 1)	118	118	*r*_P_	0.18			
		Endurance	118	118	OF activity	118	118	*r*_P_	0.02			
		Endurance	118	118	OF max speed	118	118	*r*_P_	0.08			
		Endurance	118	118	OF avg speed	118	118	*r*_P_	−0.05			
		Endurance	118	118	OF exploration	118	118	*r*_P_	0.09			
	*Bufo calamita* (NE environment)^6^	Sprint speed	152	152	OF latency (× − 1)	152	152	*r*_P_	−0.04			
		Sprint speed	152	152	OF activity	152	152	*r*_P_	0.14			
		Sprint speed	152	152	OF max speed	152	152	*r*_P_	0.11			
		Sprint speed	152	152	OF avg speed	152	152	*r*_P_	0.12			
		Sprint speed	152	152	OF exploration	152	152	*r*_P_	−0.02			
		Endurance	152	152	OF latency (× − 1)	152	152	*r*_P_	−0.04			
		Endurance	152	152	OF activity	152	152	*r*_P_	0.06			
		Endurance	152	152	OF max speed	152	152	*r*_P_	0.01			
		Endurance	152	152	OF avg speed	152	152	*r*_P_	−0.06			
		Endurance	152	152	OF exploration	152	152	*r*_P_	−0.03			
Lizards												
	*Phrynocephalus vlangalii* (F)	Sprint speed	18	18	Prob. of taking refuge (× − 1)	18	18	^1^ *r*_P_	**0.93 ± 0.03**			Qi *et al*. (2014)
	*Phrynocephalus vlangalii* (M)	Sprint speed	26	26	Prob. of taking refuge (× − 1)	26	26	^1^ *r*_P_	**−0.54 ± 0.14**			
	*Phrynocephalus vlangalii* (pooled)	Sprint speed	44	44	Flight initiation distance	44	44	*r*_P_	NS			
		Sprint speed	44	44	Flight distance	44	44	*r*_P_	NS			
		Endurance	44	44	Prob. of taking refuge (× − 1)	44	44	*r*_P_	NS			
		Endurance	44	44	Flight initiation distance	44	44	*r*_P_	NS			
		Endurance	44	44	Flight distance	44	44	*r*_P_	NS			
	*Sceloporus undulatus*	Sprint speed	38	38	Startle response	38	38	*r*_P_	**SIG**			Des Roches *et al*. (2013)
	*Aspidoscelis inornata*	Sprint speed	34	34	Startle response	34	34	*r*_P_	**SIG**			
	*Holbrookia maculata*	Sprint speed	30	30	Startle response	30	30	*r*_P_	NS			
	*Crotaphytus collaris*	Escape speed	60	60	Flight initiation distance (× − 1)	60	60	*r*_P_	**0.63 ± 0.12**			Husak (2006b)
	*Anolis cristatellus*	Endurance	50	50	Assertion displays per min	50	50	*r*_P_	**0.31 ± 0.13**			Perry *et al*. (2004)
		Endurance	50	50	Movements per min	50	50	*r*_P_	NS			
		Endurance	50	50	% time moving	50	50	*r*_P_	NS			
		Endurance	50	50	Jumps per min	50	50	*r*_P_	NS			
		Sprint speed	50	50	Assertion displays per min	50	50	*r*_P_	NS			
		Sprint speed	50	50	Movements per min	50	50	*r*_P_	NS			
		Sprint speed	50	50	% time moving	50	50	*r*_P_	NS			
		Sprint speed	50	50	Jumps per min	50	50	*r*_P_	NS			
	*Lacerta vivipara*	Endurance	600	600	Capture probability	600	600	*r*_P_	NS			Clobert *et al*. (2000)
	*Urosaurus ornatus*	Sprint speed	100	100	Dominance	100	100	*r*_P_	NS			Robson & Miles (2000)
		Endurance	100	100	Dominance	100	100	*r*_P_	NS			
	*Sceloporus occidentalis*	Sprint speed	40	40	Dominance	40	40	^2^ *r*_P_	**0.33 ± 0.16**			Garland *et al*. (1990b)
		Stamina	40	40	Dominance	40	40	^2^ *r*_P_	0.11 ± 0.16			
	*Lacerta monticola*	Sprint speed	25	25	Dominance	25	25	^1^ *r*_P_	**−0.53 ± 0.15**			López & Martín (2002)
	*Uta stansburiana*	Endurance	38	38	Dur. aggressive displays	38	38	*r*_P_	**0.49 ± 0.13**			Brandt (2003)
		Endurance	38	38	Number OF push ups	38	38	*r*_P_	0.32 ± 0.16			
	*Anolis carolinensis* (lightweight)^7^	Max. acceleration	213	213	Dominance	213	426	*r*_P_	**SIG**			Lailvaux *et al*. (2004)
		Max. jumping velocity	213	213	Dominance	213	426	*r*_P_	**SIG**			
	*Anolis carolinensis* (heavyweight)^7^	Max. acceleration	213	213	Dominance	213	426	*r*_P_	NS			
		Max. jumping velocity	213	213	Dominance	213	426	*r*_P_	NS			
	*Leiocephalus carinatus*	Endurance	45	45	Aggressive displays per min	45	173	*r*_ind_	**0.40 ± 0.17**			Diamond *et al*. (2014)
		Endurance	45	45	Movements per min	45	173	*r*_ind_	0.24 ± 0.22			
		Sprint speed	45	45	Aggressive displays per min	45	173	*r*_ind_	0.27 ± 0.22			
		Sprint speed	45	45	Movements per min	45	173	*r*_ind_	0.17 ± 0.25			
		Sprint speed	45	45	Aggressive displays per min	45	173	*r*_*e*_	−0.09 ± 0.12			
		Sprint speed	45	45	Movements per min	45	173	*r*_*e*_	−0.05 ± 0.12			
	*Phrynocephalus vlangalii*	Endurance	42	42	Time spent moving in OF	42	42	*r*_P_	**0.34 ± 0.14**			Chen *et al*. (2019)
		Endurance	42	42	Time to enter refuge	42	42	*r*_P_	−0.20 ± 0.15			
		Endurance	42	42	Risk‐taking intensity	42	42	*r*_P_	−0.15 ± 0.15			
		Sprint speed	42	42	Time in locomotion	42	42	*r*_P_	−0.03 ± 0.16			
		Sprint speed	42	42	Time to enter refuge	42	42	*r*_P_	0.02 ± 0.16			
		Sprint speed	42	42	Risk‐taking intensity	42	42	*r*_P_	0.01 ± 0.15			
	*Dipsosaurus dorsalis*	Endurance	21	42	Home range area	21	21	^1^ *r*_P_	**0.41 ± 0.19**			Singleton & Garland (2018)
	*Zootoca vivipara* (yearlings)	Sprint speed	52	52	Exploration score (PC1)	52	52	*r*_P_	**0.27**			Le Galliard *et al*. (2013)
		Sprint speed	52	52	Exploration score (PC2)	52	52	*r*_P_	0.08			
		Endurance	53	53	Exploration score (PC1)	53	53	*r*_P_	**0.25**			
		Endurance	53	53	Exploration score (PC2)	53	53	*r*_P_	0.04			
	*Zootoca vivipara* (juveniles)	Sprint speed	170	170	Exploration score (PC1)	170	170	*r*_P_	0.00			
		Sprint speed	170	170	Exploration score (PC2)	170	170	*r*_P_	0.03			
		Endurance	87	87	Exploration score (PC1)	87	87	*r*_P_	0.13			
		Endurance	87	87	Exploration score (PC2)	87	87	*r*_P_	−0.05			
	*Sceloporus woodi*	Escape speed	22	22	Flight initiation distance	22	22	*r*_P_	NS			Stiller & McBrayer (2013)
	*Gallotia galloti*	Sprint speed	39	39	Dominance	39	109	*r*_P_	NS			Huyghe *et al*. (2005)
		Acceleration	39	39	Dominance	39	109	*r*_P_	NS			
		Endurance	39	39	Dominance	39	109	*r*_P_	NS			
	*Sceloporus occidentalis*	Sprint speed	40	40	Distance per move	40	40	*r*_P_	0.07 ± 0.15			Albuquerque *et al*. (2023)
		Sprint speed	37	37	Nbr head bobs	37	37	*r*_P_	0.09 ± 0.16			
		Sprint speed	36	36	Nbr 2‐legged push‐ups	36	36	*r*_P_	−0.04 ± 0.17			
		Sprint speed	33	33	Max 4‐legged push‐ups	33	33	*r*_P_	0.06 ± 0.18			
	*Chamaeleo calyptratus*	Sprint speed	26	26	Aggressive response	26	104	^3^ *r*_P‐ind_	**−0.44 ± 0.17**			Drown *et al*. (2022)
		Sprint speed	26	26	Fleeing response (× − 1)	26	104	^3^ *r*_P‐ind_	**−0.78 ± 0.08**			
Snakes												
	*Storeria dekayi*	Swimming velocity	24	24	Death feigning dur. (× − 1)	24	24	^1^ *r*_P_	**0.84 ± 0.06**			Gerald (2008)
	*Thamnophis sirtalis*	Crawling speed	249	249	Antipredator display	249	249	^2^ *r*_P_	**0.19 ± 0.06**			Garland (1988)
		Crawling speed	249	249	Antipredator display	249	249	^3^ *r*_G_	**0.38 ± 0.19**			
		Crawling speed	249	249	Antipredator display	249	249	^3^ *r*_env_	0.11 ± 0.07			
		Endurance	249	249	Antipredator display	249	249	^2^ *r*_P_	**0.22 ± 0.06**			
		Endurance	249	249	Antipredator display	249	249	^3^ *r*_G_	0.30 ± 0.21			
		Endurance	249	249	Antipredator display	249	249	^3^ *r*_env_	**0.19 ± 0.07**			
	*Thamnophis radix*	Crawling speed	100	100	Aggressive displays	100	100	*r*_P_	**0.39**			Arnold & Bennett (1988)
Salamanders												
	*Gyrinophilus porphyriticus*	Peak swimming speed	50	50	Dispersal distance	50	50	*r*_P_	−0.48 ± 1.18			Addis *et al*. (2019)
Rodents												
	*Mus musculus*	Sprint speed	35	70	OF latency (× − 1)	35	70	^4^ *r*_ind_	**0.63 ± 0.29**			Friedman *et al*. (1992)
		Sprint speed	35	70	OF max speed	35	70	^4^ *r*_ind_	**0.68 ± 0.16**			
		Sprint speed	35	70	OF speed sd	35	70	^4^ *r*_ind_	**0.58 ± 0.21**			
		Sprint speed	35	70	OF defecations	35	70	^4^ *r*_ind_	−0.39 ± 0.28			
		Sprint speed	35	70	OF distance	35	70	^4^ *r*_ind_	0.17 ± 0.21			
		Sprint speed	35	70	Voluntary wheel running, d1	35	70	^4^ *r*_ind_	0.02 ± 0.21			
		Sprint speed	35	70	Voluntary wheel running, d7	35	70	^4^ *r*_ind_	**0.36 ± 0.20**			
		Swimming endurance	35	70	OF latency (× − 1)	35	70	^4^ *r*_ind_	0.22 ± 0.32			
		Swimming endurance	35	70	OF max speed	35	70	^4^ *r*_ind_	−0.04 ± 0.24			
		Swimming endurance	35	70	OF speed SD	35	70	^4^ *r*_ind_	−0.09 ± 0.28			
		Swimming endurance	35	70	OF defecations	35	70	^4^ *r*_ind_	0.42 ± 0.32			
		Swimming endurance	35	70	OF distance	35	70	^4^ *r*_ind_	−0.13 ± 0.23			
		Swimming endurance	35	70	Voluntary wheel running, day 1	35	70	^4^ *r*_ind_	0.07 ± 0.22			
		Swimming endurance	35	70	Voluntary wheel running, day 7	35	70	^4^ *r*_ind_	0.15 ± 0.23			
	*Mus musculus* (F)	Rotarod agility	80	80	Distance run	77	77	^1^ *r*_P_	0.17 ± 0.11			
		Rotarod agility	80	80	Time spent running	77	77	^1^ *r*_P_	0.14 ± 0.11			
		Rotarod agility	80	80	Running speed	77	77	^1^ *r*_P_	0.15 ± 0.11			
		Rotarod agility	80	80	OF left turns	76	76	^1^ *r*_P_	0.12 ± 0.12			
		Rotarod agility	80	80	OF right turns	77	77	^1^ *r*_P_	0.06 ± 0.12			
		Rotarod agility	80	80	OF dist. from centre (× − 1)	76	76	^1^ *r*_P_	−0.01 ± 0.12			
		Rotarod agility	80	80	OF latency to reach wall (× − 1)	77	77	^1^ *r*_P_	0.09 ± 0.12			
		Rotarod agility	80	80	OF time in interior	77	77	^1^ *r*_P_	−0.15 ± 0.11			
		Rotarod agility	80	80	OF defecations	78	78	^1^ *r*_P_	0.08 ± 0.11			
		Sprint speed	79	79	Distance run	76	76	^1^ *r*_P_	−0.13 ± 0.11			
		Sprint speed	79	79	Time spent running	76	76	^1^ *r*_P_	−0.01 ± 0.11			
		Sprint speed	79	79	Running speed	76	76	^1^ *r*_P_	−0.16 ± 0.11			
		Sprint speed	79	79	OF left turns	75	75	^1^ *r*_P_	−0.05 ± 0.11			
		Sprint speed	79	79	OF right turns	76	76	^1^ *r*_P_	0.06 ± 0.11			
		Sprint speed	79	79	OF dist. from centre (× − 1)	75	75	^1^ *r*_P_	0.03 ± 0.11			
		Sprint speed	79	79	OF latency to reach wall (× − 1)	76	76	^1^ *r*_P_	0.02 ± 0.11			
		Sprint speed	79	79	OF time in interior	76	76	^1^ *r*_P_	−0.02 ± 0.11			
		Sprint speed	80	80	OF defecations	78	78	^1^ *r*_P_	**0.23 ± 0.11**			Khan *et al*. (2024)
	*Mus musculus* (M)	Rotarod agility	79	79	Distance run	74	74	^1^ *r*_P_	0.14 ± 0.11			
		Rotarod agility	79	79	Time spent running	74	74	^1^ *r*_P_	0.06 ± 0.12			
		Rotarod agility	79	79	Running speed	74	74	^1^ *r*_P_	0.11 ± 0.12			
		Rotarod agility	79	79	OF left turns	74	74	^1^ *r*_P_	0.04 ± 0.12			
		Rotarod agility	79	79	OF right turns	74	74	^1^ *r*_P_	−0.06 ± 0.12			
		Rotarod agility	79	79	OF dist. from centre (× − 1)	72	72	^1^ *r*_P_	0.14 ± 0.12			
		Rotarod agility	79	79	OF latency to reach wall (× − 1)	74	74	^1^ *r*_P_	0.03 ± 0.12			
		Rotarod agility	79	79	OF time in interior	75	75	^1^ *r*_P_	−0.05 ± 0.12			
		Rotarod agility	79	79	OF defecations	75	75	^1^ *r*_P_	0.02 ± 0.12			
		Sprint speed	78	78	Distance run	74	74	^1^ *r*_P_	−0.02 ± 0.12			
		Sprint speed	78	78	Time spent running	74	74	^1^ *r*_P_	−0.02 ± 0.12			
		Sprint speed	78	78	Running speed	74	74	^1^ *r*_P_	−0.01 ± 0.12			
		Sprint speed	78	78	OF left turns	74	74	^1^ *r*_P_	−0.09 ± 0.12			
		Sprint speed	78	78	OF right turns	74	74	^1^ *r*_P_	0.06 ± 0.12			
		Sprint speed	78	78	OF dist. from centre (× − 1)	72	72	^1^ *r*_P_	−0.01 ± 0.12			
		Sprint speed	78	78	OF latency to reach wall (× − 1)	74	74	^1^ *r*_P_	−0.15 ± 0.11			
		Sprint speed	78	78	OF time in interior	75	75	^1^ *r*_P_	0.09 ± 0.11			
		Sprint speed	78	78	OF defecations	75	75	^1^ *r*_P_	0.00 ± 0.11			
	*Meriones unguiculatus*	Sprint speed	34	68	Max voluntary running speed	34	34	*r*_P_	**−0.35**			Chappell *et al*. (2007)
		Sprint speed	34	68	Distance run	34	34	*r*_P_	−0.18			
		Sprint speed	34	68	Time spent running	34	34	*r*_P_	−0.21			
	*Marmota flaviventris*	Sprint speed	187	341	Vigilance^5^	315	1237	*r*_P_	**−0.09 ± 0.04**			Blumstein *et al*. (2010)
		Sprint speed	147	238	Vigilance^5^	258	983	*r*_G_	−0.57 ± 0.28			
	*Myotomys unisulcatus*	Sprint speed	44	177	OF exploration	45	119	*r*_ind_	**−0.40 ± 0.21**			Agnani *et al*. (2020)
		Sprint speed	44	177	Docility (× − 1)	44	244	*r*_ind_	−0.28 ± 0.20			
		Sprint speed	44	177	Exploration	45	119	*r*_*e*_	−0.15 ± 0.11			
		Sprint speed	44	177	Docility (× − 1)	44	244	*r*_*e*_	−0.10 ± 0.09			
	*Tamias striatus*	Sprint speed	45	100	OF distance moved	43	64	*r*_ind_	**−0.59 ± 0.26**			Newar & Careau (2018)
		Sprint speed	45	100	OF distance moved	43	64	*r*_*e*_	**−0.53 ± 0.20**			
		Sprint speed	45	100	Docility (× − 1)	51	151	*r*_ind_	0.21 ± 0.27			
		Sprint speed	45	100	Docility (× − 1)	51	151	*r*_*e*_	0.07 ± 0.14			
		Sprint speed	45	100	Dur. in centre	43	64	*r*_ind_	0.22 ± 0.31			
		Sprint speed	45	100	Dur. in centre	43	64	*r*_*e*_	−0.12 ± 0.28			
	*Peromyscus leucopus* (lab)	Sprint speed	51	153	Voluntary wheel running	51	152	*r*_ind_	**−0.52 ± 0.15**			Agnani & Careau (2023)
		Sprint speed	51	153	Voluntary wheel running	51	152	*r*_*e*_	**0.34 ± 0.09**			
		Sprint speed	51	153	Home‐cage activity	51	153	*r*_ind_	**−0.38 ± 0.15**			
		Sprint speed	51	153	Home‐cage activity	51	153	*r*_*e*_	0.05 ± 0.10			
		Sprint speed	51	153	OF exploration	51	153	*r*_ind_	−0.27 ± 0.20			
		Sprint speed	51	153	OF exploration	51	153	*r*_*e*_	−0.06 ± 0.10			
		Swimming performance	51	152	Voluntary wheel running	51	152	*r*_ind_	**−0.37 ± 0.18**			
		Swimming performance	51	152	Home‐cage activity	51	153	*r*_ind_	−0.15 ± 0.18			
		Swimming performance	51	152	OF exploration	51	153	*r*_ind_	−0.16 ± 0.22			
	*Peromyscus leucopus* (wild)	Sprint speed	714	1665	OF distance moved	775	1874	*r*_ind_	−0.07 ± 0.08			
		Sprint speed	714	1665	OF distance moved	775	1874	*r*_*e*_	−0.03 ± 0.03			
		Sprint speed	714	1665	OF dist. from centre (× − 1)	775	1874	*r*_ind_	**−0.33 ± 0.09**			N. Bonin & V. Careau (unpublished)
		Sprint speed	714	1665	OF dist. from centre (× − 1)	775	1874	*r*_*e*_	−0.04 ± 0.03			
		Sprint speed	714	1665	OF relative area covered	771	1867	*r*_ind_	**−0.19 ± 0.09**			
		Sprint speed	714	1665	OF relative area covered	771	1867	*r*_*e*_	0.00 ± 0.03			
		Sprint speed	714	1665	OF defecations	779	1893	*r*_ind_	0.02 ± 0.09			
		Sprint speed	714	1665	OF defecations	779	1893	*r*_*e*_	0.01 ± 0.03			
		Sprint speed	714	1665	Docility (× − 1)	1174	5090	*r*_ind_	−0.13 ± 0.07			
		Sprint speed	714	1665	Docility (× − 1)	1174	5090	*r*_*e*_	0.01 ± 0.03			

^1^ Re‐calculated from a digitised version of a figure in the paper.

^2^ Re‐estimated using the raw data.

^3^ Re‐estimated using a bivariate mixed model on the raw data.

^4^ See Fig. 5 and Section IV.3 for the estimates reported in original paper.

^5^ Correlations reported in this study were not multiplied by −1 even though they involved vigilance, because sprint speed was recorded as running time adjusted for running distance [i.e. a negative correlation implies slow marmots were bold (i.e. less vigilant)].

^6^ Toads in this study were from two different cohorts, raised in a common environment (CE) and another one collected from the field (NE).

^7^ Lizards in this study showed two distinct morphs: larger ‘heavyweight’ males have relatively large heads and high bite forces for their size, whereas smaller ‘lightweight’ males have smaller heads and lower bite forces.

**Table 3:** Studies in which a performance and behavioural trait both changed significantly in response to an experimental manipulation. Only studies in which the authors found statistically significant differences in both performance and behaviour are presented (see main text). Focal treatments are indicated in bold next to the control (or second treatment). When more than two performance or behavioural traits differed between treatments, they appear as different entries; therefore, one study appears twice. Descriptions of performance measurements and behaviour are shortened but close to the original descriptions.

Taxa		Focal treatment *versus* control treatment	Performance trait	Behavioural trait	Study
Insects					
	*Acheta domesticus*	**Multi‐ingredient supplement** *vs* normal diet	Jumping distance (+)	Total distance travelled (+)	Tran *et al*. (2018)
Fish					
	*Pomacentrus amboinensis*	**Gnathids** *vs* no gnathids	C‐start response speed (−)	Routine swimming distance (−)	Allan *et al*. (2020)
	*Poecilia reticulata*	**High** *vs* low water velocity	Critical swimming speed (+)	Mean duration of sigmoid displays (+)	Nicoletto (1996)
	*Poecilia reticulata*	**High** *vs* low water velocity	Critical swimming speed (+)	Total time spent displaying (+)	
	*Trachinotus carolinus*	**Ethylene glycol exposure** *vs* control	Critical swimming speed (−)	Activity (−)	Hymel *et al*. (2002)
Amphibians					
	*Pleurodeles waltl*	**Snake kairomone**s *vs* no kairomones	Escape speed (+)	Flight initiation distance (+)	Javier Zamora‐Camacho *et al*. (2018)
Reptiles					
	*Lacerta vivipara*	**Gravid** *vs* non gravid	Running speed (−)	Flight distance (−)	Bauwens & Thoen (1981)
	*Crotaphytus collaris*	**Gravid** *vs* non gravid	Sprint speed (−)	Flight distance (−)	Husak (2006a)
	*Anolis grahami*	**Reduced** *vs* normal perch diameter	Sprint speed (−)	Frequency of jumping (+)	Losos & Irschick (1996)
	*Anolis gundlachi*	**Reduced** *vs* normal perch diameter	Sprint speed (−)	Frequency of jumping (+)	
	*Anolis lineatopus*	**Reduced** *vs* normal perch diameter	Sprint speed (−)	Frequency of jumping (+)	
	*Anolis sagrei*	**Reduced** *vs* normal perch diameter	Sprint speed (−)	Frequency of jumping (+)	

**Table 4:** Cases in which artificial selection on behaviour or performance resulted in a correlated response to selection on performance or behaviour. Only studies in which a statistically significant correlated response was found are presented. When a difference was found between only a high and a low line, the study appears as a single entry. Descriptions of performance measurements and behaviour are shortened but close to the original descriptions.

Taxa		Trait selected for	Correlated response	Study
Fish				
	*Danio rerio*	Boldness	Fast start performance	Kern *et al*. (2016)
Rodents				
	*Mus domesticus*	Voluntary wheel running	Endurance	Meek *et al*. (2009)
	*Rattus rattus*	Endurance	Voluntary wheel running	Waters *et al*. (2008)
		Endurance	Novel environment activity	Waters *et al*. (2010)

### (1) Extraction of effect sizes

For each study, we report the level at which the relationship was observed (i.e. phenotypic, among‐ or within‐individual levels) and, when available, indicate the strength of association reported. We chose to retain the vocabulary used by the authors to describe their measurement of performance and of behaviour. In some cases, the correlation estimate was multiplied by −1, such that a positive correlation indicates that high locomotor performance is associated with higher levels of behavioural activity, exploration, and boldness (i.e. higher risk taking). The multiplication by −1 was done whenever the behavioural trait was recorded as a latency to move, flight initiation distance, startle duration, probability of taking refuge, death feigning duration, vigilance, docility, distance from centre, and thigmotaxis (i.e. for 32 correlations in Table 2, indicated by × −1, and one non‐significant association indicated as NS).

Some studies tested for an association between a performance and a behavioural trait, but did not report the actual correlation estimate (Clobert *et al*., 2000; Des Roches *et al*., 2013; Huyghe *et al*., 2005; Kodric‐Brown & Nicoletto, 1993; Lailvaux *et al*., 2004; Le Roy & Seebacher, 2018; Robson & Miles, 2000; Stiller & McBrayer, 2013). These studies are still compiled in Table 2, without the correlation estimate. Some studies reported the correlation estimate without an uncertainty; whenever possible, we re‐calculated the correlation estimate ± SEM, either by digitising the figures [e.g. Figure 4 in Gerald (2008); fig. 1 in Nelson & Formanowicz (2005); figs 1 and 2a in López & Martín (2002); fig. 4b in Singleton & Garland (2018); Fig. 2 in Perry *et al*. (2004); fig. 2b in Qi *et al*. (2014)] or accessing the original raw data (Bredeweg *et al*., 2019; Chen *et al*., 2019; Courtene‐Jones & Briffa, 2023; Drown, Liebl & Anderson, 2022; Garland, 1988; Garland, Hankins & Huey, 1990b; Kasumovic & Seebacher, 2018; Khan *et al*., 2024).

## III. RESULTS

### (1) Correlations between performance and behaviour

We found 45 studies that tested for a link between performance and behavioural traits, of which 33 found at least one statistically significant association (Table 2). Most studies were conducted on reptiles (all lizards and snakes) and mammals (all rodents), but Table 2 also includes many studies on taxa such as arthropods, fishes, and amphibians. The performance traits mostly consist of classical measures of locomotor performance (e.g. speed, acceleration, endurance) in relation to various behaviours, such as activity, exploration, boldness, and aggressiveness, but also behavioural traits like dominance, lateralisation, and thanatosis. Overall, sample sizes ranged from 12 to 817 individuals measured and the number of repeated measures per individual ranged from 1 to 4 for performance and 1 to 8 for behavioural traits.

**Fig. 3:**
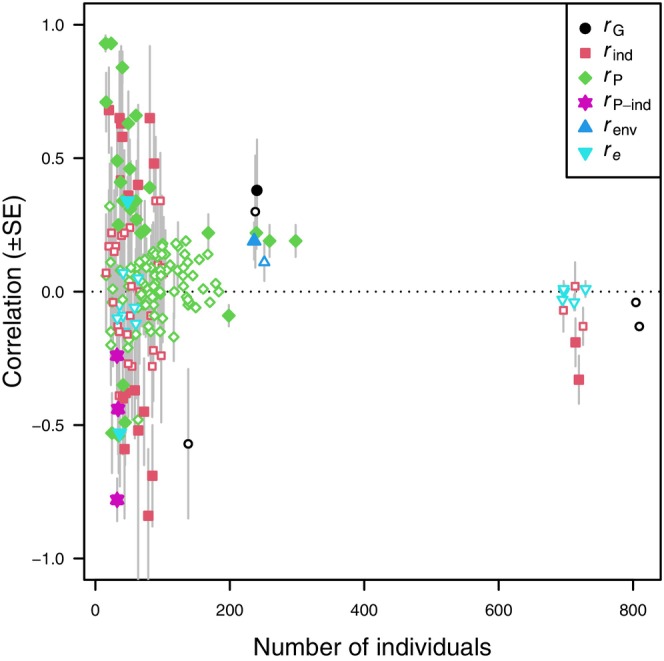
Correlations between performance and behaviour. Correlation estimates between performance and behavioural traits (i.e. from Table 2) as function of the number of individuals measured (i.e. sample size). The type of correlation (see text for explanations) is shown by different symbols: genetic correlations (*r*_G_; black dots), among‐individual correlations (*r*_ind_; red squares), phenotypic correlations (*r*_P_; green lozenges), correlations between the phenotypic variation in one trait and the among‐individual variation in the other (*r*_P‐ind_; purple stars), environmental correlations (*r*_env_; blue up‐triangles), and residual (usually within‐individual) correlations (*r*_*e*_; magenta down‐triangles). Solid symbols indicate estimates that differ statistically from zero. When needed, estimates were multiplied by −1 such that a positive correlation indicates higher performance is associated with higher levels of locomotor activity (movement) exploration, and boldness (see Table 2).

We were able to extract many effect sizes, but some studies reported associations between performance and behaviour for which effect sizes were not included and could not be calculated. Most (34 out of 56) of the significant correlations were quantified at the phenotypic level (or between the phenotypic variation in one trait and the among‐individual variation in the other), whereas only 18 were observed at the among‐individual level, three at the residual (within‐individual or environmental) level, and only one significant correlation was reported at the genetic level. Figure 3 indicates important heterogeneity in effect sizes, with many high‐correlation estimates at low sample sizes, and moderate‐ to low‐correlation estimates as sample size increased. Although it would be interesting to test for the presence of publication bias, we are unsure whether this is relevant here because we calculated many of the estimates using digitised graphs, published data sets, or by contacting authors.

### (2) Correlated plasticity in performance and behaviour

We found eight studies that reported evidence of correlated plasticity in performance and behaviour, for a total of 12 associations in insects, fish, amphibians, and reptiles (Table 3). Correlated phenotypic plasticity in performance and behaviour occurred in response to various environments and treatments, including parasites, contaminants, predator cues, diet, and substrate. The performance traits measured included different measurements of endurance, speed or jumping distance, whereas behaviours captured aspects of activity, aggressiveness, and risk‐taking. Perhaps the most striking examples of correlated plasticity in performance and behaviour come from two studies comparing gravid *versus* non‐gravid female lizards. In both Bauwens & Thoen (1981) and Husak (2006a), gravid females reduced running speed or maximal sprint speed measured in the laboratory but also fled from a putative human predator over a shorter distance than non‐gravid females (i.e. they ran shorter distances), suggesting that gravid females compensated for reduced locomotor capacity by staying closer to refugia. In contrast to such compensatory changes in performance and behaviour, Javier Zamora‐Camacho, García‐Astilleros & Aragón (2018) showed that when exposed to snake kairomones, newt larvae were both more responsive to an approaching predator model (i.e. a longer flight initiation distance) and displayed greater escape speed. Thus, there are examples of correlated plastic changes in performance and behaviour occurring in both compensatory and additive ways.

### (3) Correlated responses to selection on performance or behaviour

We only found one artificial selection experiment on performance that produced two significant correlated responses in behaviour (Table 4). In earlier work, Koch & Britton (2001) created two lines of rats using bidirectional artificial selection for forced treadmill running capacity such that after 10 generations, rats bred as low‐capacity runners (LCR) and high‐capacity runners (HCR) differed by 317% in treadmill running capacity. Waters *et al*. (2008) showed that HCR rats voluntarily ran on wheels 33% greater distance per day compared to LCR rats. Moreover, Waters *et al*. (2010) showed that after 1 h of restraint stress and a 24‐h recovery period, HCR rats spent less time in the open arms of an elevated plus maze test, crossed fewer lines and reared less often than LCR rats during a novel environment test.

We found two studies in which artificial selection on behaviour produced significant correlated responses in performance (Table 4). After seven generations of artificial selection, a zebrafish (*Danio rerio*) line selected for high boldness had higher fast‐start escape performance than another line selected for low boldness (Kern *et al*., 2016). The other selection experiment on behaviour that produced a correlated changed in performance was conducted on house mice (*Mus musculus*). Four replicate ‘high runner’ (HR) lines (selected for high voluntary wheel running) had significantly higher endurance capacity than the four replicate control lines (Meek *et al*., 2009). Khan *et al*. (2024) found, however, that the HR mice at generation 22 of the experiment did not differ from the control mice in terms of sprint speed or rota‐rod performance (i.e. agility, as measured by the ability to remain in balance on a suspended rod rotating on itself; Jones & Roberts, 1968).

Finally, we highlight an interesting and well replicated, bi‐directional selection experiment on behaviour and performance that almost qualified, but the performance measurements did not quite meet our definition. The experiment was conducted on bean beetles (*Callosobruchus chinensis*) by Ohno & Miyatake (2007), in which they applied selection on death‐feigning behaviour and observed a correlated response in flight ‘ability’, and *vice versa*. Although one would think flying ‘ability’ is the same as ‘performance’, their measure of flight ‘ability’ was completely voluntary in that they did not attempt to apply any motivation. This study illustrates how it can be difficult to decide whether a trait represents behaviour or performance, but in any case, we recommend that researchers are careful and consistent with their terminology and always include a sentence or two explaining why call a measured variable qualifies as behaviour *versus* performance.

## IV. DISCUSSION

Overall, we found many studies that report statistically significant correlations (Table 2), correlated plasticity (Table 3), and correlated responses (Table 4) to selection between performance and behaviour. A phylogenetically informed meta‐analysis (Adams, 2008) will be needed to explore the heterogeneity in effect sizes shown in Table 2 more thoroughly. In the absence of such analyses, we use the studies reviewed in Tables 2, 3, 4, to discuss current empirical support for the compensation and co‐specialisation hypotheses and make recommendations to guide future research on locomotor performance and behaviour, including (*i*) paying more attention to sampling bias and sources of variation in performance across levels, (*ii*) adopting a (co)variance partitioning approach, (*iii*) an increased focus on quantitative genetic studies, and (*iv*) developing the behaviour–performance continuum.

### (1) The compensation and co‐specialisation hypotheses

**Fig. 4:**
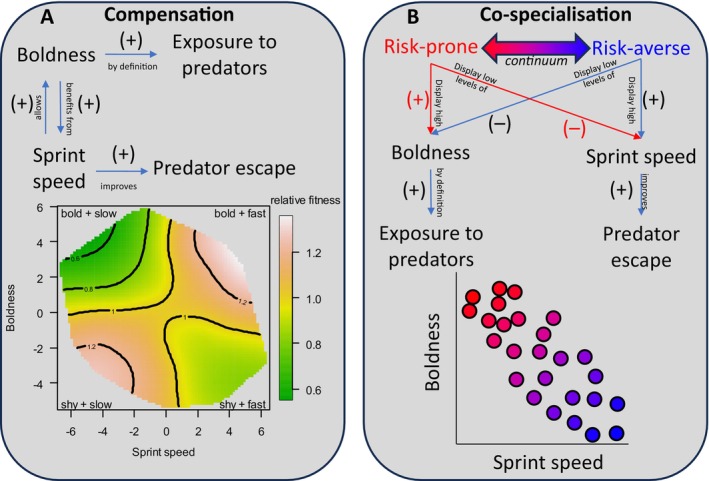
The compensation and co‐specialisation hypotheses. Hypothetical mechanisms that could cause correlations between performance and behavioural traits. In A, trait compensation arises from correlational selection on boldness and sprint speed, yielding two trait combinations (bold+fast and shy+slow) with high relative fitness and two (bold+slow and shy+fast) with low relative fitness. In B, trait co‐specialisation arises from variation in sensory and cognitive abilities needed to detect predators, which causes variation from a risk‐averse (shy and fast) to risk‐prone (bold and slow) gradient. See text for further discussion.

DeWitt, Sih & Hucko (1999) originally proposed two hypotheses to explain the links between morphological defences and anti‐predator behaviour – the compensation and co‐specialisation hypotheses. More recently these two hypotheses have been used in studies of links between behaviour and performance, with the assumptions that (*i*) high levels of activity, exploration, aggressiveness, and boldness lead to a higher predation risk and (*ii*) performance traits such as locomotor speed, agility, and strength increase the probability of escaping predators. According to the compensation hypothesis (Fig. 4A), individuals with greater locomotor performance can ‘afford’ to take more risks (or, put another way, individuals that expose themselves to more risky situations should compensate with greater abilities to evade risky situations). According to the co‐specialisation hypothesis (Fig. 4B), both performance and behavioural traits additively reduce predation risk, such that co‐specialisation should generate negative relationships between performance and behaviour. In other words, risk‐averse individuals, who are shy and hence less exposed to predators, are also expected to be equipped with better locomotor machinery to escape predator encounters, yielding a negative correlation between boldness and sprint speed.

We note that, for some of the studies included in this review, it was not entirely clear if the association reported should be interpreted as supportive of the compensation or co‐specialisation hypothesis. This is because the reasoning behind the two hypotheses is clearly articulated around predation risk (see above, Fig. 4), but some of the behaviours measured are not directly relevant in an anti‐predator context (e.g. territoriality, aggressiveness, and lateralisation). In the strictest sense, the only relevant measures are those capturing aspects of boldness, but we nevertheless included all behaviours in our interpretation, with the assumption that expressing higher locomotor activity (movement) exposes animals to higher predation risk.

Overall, the empirical support for the compensation hypothesis seems solid. Of the 56 significant correlations compiled in Table 2, 35 were supportive of the compensation hypothesis [note that the two significant relationships in Lailvaux *et al*. (2004) were in support of compensation, and the two significant relationships in Des Roches *et al*. (2013) were not]. Of the 12 associations reported in Table 3, most (eight, or 66%) were in support of the compensation hypothesis. Finally, of the four correlated responses documented in Table 4, three were in support of the compensation hypothesis.

To our knowledge, the mechanisms that underlie trait compensation have not yet been clearly identified, but various mechanisms could be involved. With respect to ultimate causation, trait compensation could result from correlational selection (i.e. non‐linear selection on trait combinations: Sinervo & Svensson, 2002). As a simple way to illustrate correlational selection on behaviour and performance, let us consider boldness and sprint speed in the context of predator–prey interactions [Fig. 4; see also Brodie (1989, 1992) and Ohno & Miyatake (2007)]. By definition, bold individuals take more risks and should therefore be more exposed to predators, in which case it would be advantageous to be well equipped with locomotor abilities (and the requisite underlying machinery, e.g. biochemistry, morphology) that facilitate escape from predator attacks. By contrast, being able to run fast might not be as important for shy individuals who take fewer risks and are presumably less exposed to predators. In this case, behaviour is shielding or acting as a filter between selection and performance (Garland, Bennett & Daniels, 1990a; Garland & Losos, 1994), and it would be advantageous for shy individuals to save the cost of building and maintaining the machinery required for higher sprint speed. Therefore, it is easy to imagine how these two combinations of boldness and sprint speed (bold and fast, or shy and slow) could yield higher fitness than the other two combinations (bold and slow, or shy and fast; Fig. 4A). Such correlational selection, in which the form of selection on one trait depends on the expression of another, if continued for many generations, should result in a positive (genetic) correlation between performance and behaviour (Garland *et al*., 2022). Several workers have emphasised that combinations of behavioural and performance traits are likely under correlational selection (Brodie, 1992; Forsman & Appelqvist, 1998; Garland, 1994; Ohno & Miyatake, 2007; Sinervo & Clobert, 2003).

Although support for the compensation hypothesis seems strong, many significant correlations were of the opposite sign, instead lending support for the co‐specialisation hypothesis. Indeed, 21 of the 56 significant correlations compiled in Table 2 were in support of the co‐specialisation hypothesis [note that the two significant relationships in Des Roches *et al*. (2013) were in support of co‐specialisation]. Moreover, four of the 12 associations reported in Table 3 were in support of the co‐specialisation hypothesis. It is relatively easy to imagine how selection could favour ‘shy and fast’ phenotypes, because such individuals would be well equipped both to avoid and to run away from predators. However, the co‐specialisation entails the existence of ‘bold and slow’ phenotypes, a trait combination that is harder to explain from an adaptive perspective. In fact, it is hard to imagine a situation in which selection would favour slow sprint speed in any trait combination – shy animals probably rarely need to use sprint speed, but, if and when they do, being fast should be favoured. And if bold animals need to use sprint speed more often, then how can they survive if they are slow runners? Perhaps the ‘bold and slow’ phenotypes are maladaptive, and arise from occasional random mutations.

In their original study, DeWitt *et al*. (1999) suggested two mechanisms underlying co‐specialisation between morphological defences and anti‐predator behaviours. The first potential mechanism is developmental plasticity, in which variation in the environment would generate individual (co)variation in both behaviour and locomotor performance. For example, predator encounters may result in stronger anti‐predator behaviour and higher motivation to perform in experienced *versus* naïve individuals. We note that correlated developmental plasticity can itself be a result of past selection (Scheiner, 1993). In any case, correlated plastic responses, unless statistically controlled for, may generate a negative correlation between boldness and sprint speed. The second mechanism for co‐specialisation involves variation in the ability to detect predators, which may arise from sensory and cognitive functions. In this interesting possibility, some individuals might appear as prone to predation risk (i.e. bold and slow) simply because they are bad at detecting predators (DeWitt, 1998). By contrast, risk‐averse individuals may be better at detecting predators and evaluating predation risk, which would explain why they behave more shyly and are better equipped to deal with predator encounters (e.g. they run faster).

Although the above remains largely speculative, in some contexts it may be possible to design experiments to verify if experience and/or sensory abilities are at play. For example, one could manipulate prior experience with predators and see if it generates correlated plastic responses in performance and behaviour (alternatively, individual differences in prior exposure to predators could be controlled for statistically to see if it explains part of the correlation between performance and behaviour). Another way would be to manipulate conditions that make predators more (or less) easy to detect; if individual differences in sensory ability to detect predators generates co‐specialisation, then making predators easily (or impossibly) detectable should reduce the negative relationship between behaviour and performance.

### (2) Sampling bias and sources of variation in performance across levels

Based on our reading of the reviewed studies, we noticed that it is common practice to exclude individuals based on their phenotype. For example, many studies explicitly mention excluding some individuals because they refused to cooperate in the performance trials. Similarly, some individuals were excluded because they did not express the behaviour of interest. In Gerald (2008), for example, two out of 26 individuals had to be excluded because they did not feign death, so their behaviour could not be measured. Future studies should pay more attention to exclusions and verify whether they occur at random or not, and modify protocols accordingly. For example, a comparison of the excluded *versus* included individuals could be made with respect to traits that were measured on all individuals; if the excluded individuals do not differ from the included individuals in any other aspect than performance (or behaviour), then this suggests sampling bias is unlikely to be responsible for the association (or lack thereof) between performance and behaviour in the sample of retained individuals.

Most studies in Table 2 contain repeated measures of performance, but only retained a single ‘personal best’ value per individual, which is likely to introduce bias (Adolph & Hardin, 2007; Adolph & Pickering, 2008; Careau & Wilson, 2017a; Head *et al*., 2012). Of course, the goal is to study organismal performance, and therefore trials that are clearly sub‐maximal and those in which animals exhibit odd behaviours, such as jumping out of a racetrack, or not performing as expected in the test, can, and arguably should, still be removed (see also Losos *et al*., 2002). Even within the remaining retained trials, however, there should be some variation in motivation, so the question is how much variation in performance measures is due to variation in motivation. In principle, the answer should be zero (e.g. see definition in Table 1 and Careau & Garland, 2012). Here, we propose that properly accounting for the hierarchical nature of performance data might represent a partial statistical solution to an inherently methodological challenge and allow one to estimate the extent of variation in performance measurements due to motivation.

A common way to measure performance is to test animals over multiple days (‘sessions’) and conduct multiple trials within each test day. When performance is measured in this way, it becomes possible to partition variation at three distinct levels: (*i*) among individuals; (*ii*) among tests within individuals; and (*iii*) among trials within tests (days or sessions). In some cases, multiple performance measurements are taken within each trial. For example, one could divide a 5‐m long racetrack into five 1‐m long sections, and extract a speed measurement for each section of the racetrack. In this case, it is possible to quantify a variance estimate at the trial level separately from the variation that occurs from one section of the track to another. Within‐ and among‐trial variance (i.e. the variation across sections of a racetrack and among repeated trials made on an individual on a given day) should capture variation due to measurement error and ‘transient’ fluctuations that are supposedly irrelevant to the actual performance trait of interest (Ponzi *et al*., 2018). Interestingly, the transient effects may include such effects as motivation, fatigue, and habituation. Hence, the variation observed within and among successive trials (within tests) may become particularly relevant to consider in light of the challenge posed by variation in motivation. In an analysis of the raw performance measurements, the effect of trial order (within tests) will indicate some combination of learning the challenge (e.g. to run in straight line along a racetrack), habituation (e.g. reduced response to whatever methods are being used to maximise motivation), physical conditioning (i.e. positive training effects that can occur within days), and/or fatigue (i.e. decreased ability to perform across repeated trials, few studies are available see also Bennett, 1980). Although it might seem important to include trial order in the analysis and report its effect size and % variance explained, interpreting what it means is difficult because it potentially confounds multiple variables (learning, habituation, conditioning, fatigue, see above). In any case, once trial sequence is controlled for, the remaining variation among trials within tests would most likely reflect variation in motivation (in addition to measurement error). It then becomes interesting to compare the amount of residual variance (which includes motivation and measurement error) to the among‐ and within‐individual variation in performance. Such partitioned variance estimates are starting to appear in the literature (Table 5), along with short‐ and long‐term repeatability estimates (Araya‐Ajoy, Mathot & Dingemanse, 2015; see also Van Berkum et al., 1989; Huey et al., 1990) of performance, but more estimates are needed before patterns may emerge. If the importance of motivation differs across levels, however, then one could expect differences in the sign and strength of relationship with behaviour (which, ultimately, is more strongly determined by motivation).

**Table 5:** Proportion of variance in raw measurements of (A) sprint speed, (B) bite force, and (C) grip strength at three separate hierarchical levels: among individuals, among tests (within individuals), and among trials (within tests, corresponding to residual level). In cases where locomotor speed is measured separately across different sections of the racetrack, a fourth level of variance can be estimated at the within‐trial level (i.e. variation in speed from one section of the racetrack to another, corresponding to residual level). NE = not estimable because one measurement per trial was retained. NA = not applicable because bite force and grip strength trials typical return a single measure.

Trait	Species		Sex	Proportion of variance			Among ind.	Study
				Within trials	Among trials	Among tests		
(A) Sprint speed								
	White‐footed mice (wild)	*Peromyscus leucopus*	pooled	NE	0.64	0.19	0.17	Berberi & Careau (2019)
	Green anole lizard	*Anolis carolinensis*	pooled	NE	0.54	0.27	0.19	Lailvaux *et al*. (2019)
	Karoo bush rats (wild)	*Myotomys unisulcatus*	pooled	0.49	0.06	0.10	0.34	Agnani *et al*. (2020)
	White‐footed mice (captive)	*Peromyscus leucopus*	pooled	0.37	0.05	0.23	0.35	Agnani & Careau (2023)
(B) Bite force								
	Green anole lizard	*Anolis carolinensis*	males	NA	0.44	0.21	0.35	Lailvaux *et al*. (2019)
	Green anole lizard	*Anolis carolinensis*	females	NA	0.28	0.33	0.39	Lailvaux *et al*. (2019)
(C) Grip strength								
	White‐footed mice (wild)	*Peromyscus leucopus*	pooled	NA	0.54	0.27	0.19	Berberi & Careau (2019)

### (3) Time to move beyond phenotypic correlations

The vast majority of performance–behaviour correlations have been estimated at the phenotypic level (see Table 2). These estimates, however, can be misleading because the phenotypic correlation (*r*_P_) can be greatly attenuated due to measurement error, resulting in an effect size being closer to 0 than it really is (Trafimow, 2016). Furthermore, a *r*_P_ based on single measurements per individual can be close to zero not because the studied traits are independent, but because the *r*_P_ represents a mix of among‐ and within‐individual (co)variances that are of comparable strength but in opposite directions (Careau & Wilson, 2017a). When multiple paired measurements are taken per individual, it is possible to partition (co)variances and estimate the among‐individual correlation (*r*_ind_) separately from the within‐individual (residual) correlation (*r*_*e*_) (Dingemanse & Dochtermann, 2013). The *r*_ind_ represents the association among individuals (i.e. within a sampled population, are bolder individuals also faster sprinters?). By contrast, the *r*_*e*_ represents the association within individuals (e.g. if an individual is bolder according to its own average on a given day, is it also faster?). Such studies, in which *r*_P_ is partitioned into *r*_ind_ and *r*_*e*_, are much needed to understand better the links between performance and behaviour, especially when correlations can be of opposite directions at those two levels (e.g. Agnani & Careau, 2023) [for a comparable situation, but applied to (co)variation at the phylogenetic *versus* independent levels, see Halliwell, Holland & Yates (2025)].

Only a small fraction of the studies included in Table 2 have partitioned (co)variances to estimate the *r*_ind_ and *r*_*e*_ between performance and behaviour. Upon examination of the structure of the data collected, we believe that many studies would permit a (co)variance partitioning approach (e.g. Arnold & Bennett, 1988; Brandt, 2003; Drown *et al*., 2022; Friedman, Garland & Dohm, 1992; Garland, 1988; Gerald, 2008; Lailvaux *et al*., 2004; Reim *et al*., 2019). Hence, re‐analysing published data sets with multivariate mixed models would be an easy and potentially rewarding way to gain a more complete picture of the association between performance and behaviour. Below, we include a re‐analysis of a previously published study (Friedman *et al*., 1992) that contained repeated measurements.

**Fig. 5:**
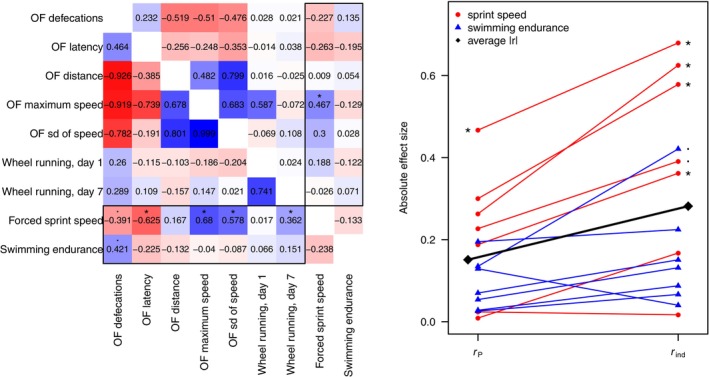
Correlation matrices between two locomotor performance traits (sprint speed and swimming endurance), five open‐field (OF) test variables, and two voluntary wheel‐running measures (distance run on day 1 and 7), reported as phenotypic correlations (*r*_P_; upper right triangle of correlations in left panel; using one measure per individual) in Friedman *et al*. (1992) and as among‐individual correlations (*r*_ind_; lower left triangle of correlations in left panel; estimated with a multivariate mixed model that included all repeated measures). Asterisks (*) and dots (.) in both panels indicate *P* < 0.05 and *P* < 0.1 estimated using profile likelihoods (Wolak, 2012).

Friedman *et al*. (1992) repeatedly measured several locomotor performance and behavioural traits in 35 adult male house mice (*Mus musculus*), including forced sprint speed, swimming endurance, five behaviours in an open‐field test (defecations, movement latency, distance, maximum speed, SD of speed), and voluntary wheel running (over 7 days). Original data files and SPSS code were kindly provided by the authors for replication and re‐analysis. Overall, the strength of the relationships between performance and behavioural measures was weak (mean |*r*| = 0.151, Fig. 5), and only one of the 14 performance‐behaviour relationships was significantly different from zero (i.e. *r* = 0.467 between forced sprint speed and the maximum speed voluntarily expressed in the open‐field test).

While the Friedman *et al*. (1992) study was innovative, standard statistical software at the time did not easily permit a full consideration of the repeated‐measures data set, at least not without violating some model assumptions (e.g. independent error). With their primary goal of testing ‘for correlations between putatively behavioural and physiological measures of locomotor capacities’ (p. 101), Friedman *et al*. (1992) retained the higher of two replicate values for each performance trait. Moreover, the open‐field test with the shortest movement latency was retained and voluntary wheel running on days 1 and 7 were considered as separate traits. The resulting phenotypic correlations, however, were most likely attenuated by a certain degree of measurement error and within‐individual variation, which represented about half of the phenotypic variation in the traits (i.e. the average repeatability was *R* = 0.56, range 0.29–0.73).

We re‐analysed data from Friedman *et al*. (1992) to gain insight regarding the issue of correlation attenuation and to illustrate the (co)variance partitioning approach. After replicating repeatability values using univariate mixed models, we ran a multivariate mixed model that included all of the repeated measurements made on each individual. Test sequence was fitted as a fixed effect to account for mean differences between the first and second tests. Individual identity (ID) was also included as a random effect fitted as an unstructured correlation matrix to estimate among‐individual variance and all possible among‐individual correlations (*r*_ind_). On average, the *r*_ind_ between performance and behavioural traits was noticeably stronger (|*r*_ind_| = 0.282; black diamonds in Fig. 5). Four among‐individual correlations were significantly different from zero and two more were approaching significance (Fig. 5).

Therefore, reassuringly, our reanalysis of data from Friedman *et al*. (1992) using the (co)variance partitioning approach yielded relatively similar results, and none of their conclusions would change dramatically. However, the overall difference in average effect sizes (|*r*_P_| *versus* |*r*_ind_| of 0.151 *versus* 0.282) most likely represents the level of attenuation that was present in the originally reported estimates (Fig. 5).

Clearly (co)variance partitioning represents an efficient and powerful tool for testing trait correlations while accounting for the hierarchical structure of the data. Moreover, here we only focused on *r*_ind_, but the approach can be extended to explore patterns of within‐individual changes (phenotypic plasticity) over repeated tests. Given the number of studies that report correlated changes in performance and behaviour in response to changes in the environment (Table 3), correlated plasticity may be expected to generate significant within‐individual covariance between performance and behaviour. The (co)variance partitioning approach has proved useful not only for detecting previously obfuscated patterns of trait correlations (Careau & Wilson, 2017a,b), but also for planning experimental designs that require repeated sampling of multiple traits (Dingemanse & Dochtermann, 2013). For example, studies on animal personality and behavioural syndromes should (*i*) include repeated measures, (*ii*) report the among‐ and within‐individual (co)variance, and (*iii*) avoid applying two‐step (‘stats on stats’) analyses (Dingemanse & Wright, 2020). By extension, these criteria apply to any study wanting to test for associations between behavioural and performance traits.

Many logistical challenges arise when attempting to collect paired data on multiple performance and behavioural traits, which impose constraints on achieving a fully multivariate and hierarchical data set. For example, a performance test on one day is likely to cause physiological changes that affect subsequent measures of performance or behaviour for at least a day. Hence, most investigators only measure one trait (whether performance or behaviour) on a given day. If both traits have repeated measures (on different days), we can still estimate *r*_ind_ but not *r*_*e*_ (Dingemanse & Dochtermann, 2013). This is because the time elapsed between the measurements of the two traits increases the chance that micro‐environmental factors that were present on a given day when trait *x* was measured are no longer present when trait *y* was measured. In practice, the observations cannot be paired on the same line in the data set, which prevents statistical estimation of a covariance at the residual level.

Of course, one could take the reasoning above *ad infinitum*, to a point where it becomes impossible to pair any measurement because one cannot measure behaviour and performance at exactly the same time. Determining which measurements can be paired (or not) should be considered relative to the time span of the data collection and the biology and environment of the organisms under study. In a long‐lived temperate bird, for example, it might be reasonable to pair behavioural and performance measurements made on separate weeks within same nesting season (but repeated across years). In short‐lived *Drosophila* flies, however, it could be hard to justify pairing measurements taken more than a day apart. One possible solution would be to design a protocol in which the first part of the test quantifies behaviour, immediately followed by a forced‐exercise test to measure performance, as done in Kasumovic & Seebacher (2018). In this case, the behavioural and performance measurements are made inside the same trial and so at the same time and should be paired for analysis. In other cases, there will always be a certain degree of subjectivity involved in determining which measurements should be paired (or not). At one extreme, when deciding to pair measurements that were made far apart, the microenvironmental factors that apply to one measurement (e.g. behaviour) would be completely different than those that apply to the other measurement (e.g. performance), such that the resulting within‐individual correlation will most likely be estimated at zero. At the other extreme, when deciding that two traits should not be paired, then the residual correlation cannot be estimated, and this is the equivalent of assuming the residual covariance is zero.

In some cases, we have a mixture of repeatedly measured traits and singly measured traits. For example, Courtene‐Jones & Briffa (2023) and Drown *et al*. (2022) collected many repeat behavioural observations, but a single performance measurement per individual. We can still use multivariate mixed models to estimate the correlation between the repeatable component of the behavioural variation and the (phenotypic) variation in performance (*r*_P‐ind_). Some power is then gained because *r*_P‐ind_ is estimated separately from the measurement error (and within‐individual changes) in the repeatedly measured variables. [Note: this situation also applies to many lower‐level traits that can only be measured once, e.g. heart mass obtained by dissection.] The model fitting required to estimate *r*_P‐ind_ involves constraining the residual variance component for the singly measured trait to a value of 0. As pointed out by Morrissey *et al*. (2012), this does not imply that we can estimate among‐individual variance (*V*_ind_) in a singly measured trait (e.g. lifetime reproductive success), but this constraint in the model allows the statistical estimation of the biologically interesting relationship between the phenotypic variation in the singly measured trait and the repeatable component of variation in the other. To illustrate this approach, we accessed online data from both Courtene‐Jones & Briffa (2023) and Drown *et al*. (2022) and recalculated *r*_P‐ind_ (as reported in Table 2). The code (Data S1) and data (Data S2) for Courtene‐Jones & Briffa (2023) are provided as online Supporting Information.

### (4) Quantitative genetic studies

Despite some early efforts to study locomotor performance from the perspective of quantitative genetics (Brodie, 1989; Dohm, Hayes & Garland, 1996; Garland, 1988; Sorci *et al*., 1995), few studies are available. Associations between performance and behaviour can occur at multiple levels, including the genetic, phenotypic, among‐individual, and residual levels. A genetic correlation indicates the degree to which two traits share a genetic basis (Dickerson, 1955; Roff, 1995) due primarily to pleiotropy and linkage disequilibrium, and is a key parameter of quantitative genetics with important evolutionary consequences (e.g. constraint) (Arnold, 1987, 2023). Quantifying genetic correlations, however, requires large sample sizes and measurements on individuals with a known pedigree or *via* a planned breeding experiment.

We only found evidence of one significant genetic correlation between performance and behaviour: crawling speed was positively genetically correlated with antipredator displays in garter snakes (Garland, 1988). Moreover, Blumstein *et al*. (2010) found that escape speed tended to be negatively genetically correlated with vigilance in wild marmots (Blumstein *et al*., 2010). Another study reported weak negative genetic covariances between jumping distance and power and calling effort in crickets (Lailvaux, Hall & Brooks, 2010), but the effect sizes were weak and not significantly different from zero (Table 2). Evidence from selection experiments lends further support for trait association at the genetic level (Table 4). Indeed, correlated changes in performance in response to selection on behaviour (or any other trait, for that matter) indicate a genetic correlation. Clearly, we need more quantitative genetic studies on locomotor performance and behaviour – a first step in that direction will be to apply (co)variance partitioning approaches to separate sources of (co)variances at the among‐ *versus* within‐individual levels (see Section IV.4). Given that genetic correlations should often occur through developmental or physiological interconnections (i.e. pleiotropy), an important avenue for future research will also be to look for proximate, mechanistic explanations (i.e. a common neuronal, hormonal, or metabolic system) to the observed links between performance and behavioural traits (see also Careau and Garland, 2012).

### (5) The behaviour–performance continuum

Although great care must be taken to make sure performance is measured on a maximally motivated animal, the possibility that variation in motivation generates part of the variability observed in the performance measurement always remains. To the extent that this occurs, then covariance with behaviour might be due to a methodological bias and/or individual differences in stress coping styles (Koolhaas *et al*., 2007), pain tolerance or other factors that might determine the form and intensity of the reaction to the stimulus used (human approach, electric shock, etc.). Given that motivational circuits in the brain can also interact with many other physiological or neurobiological pathways, such as fear (Stankowich & Blumstein, 2005), pain (Bank *et al*., 2013) or hunger (Burnett *et al*., 2016), studies in which performance is measured independently of an animal's reaction towards a human threat (i.e. not relying on escape) are greatly needed. Even after partitioning correlations and using a multilevel approach, motivation can remain a problem.

Perhaps a way forward is to include multiple measurements of both behaviour and performance that vary in how much they are putatively influenced by stress and motivation. This could be done, for example, by placing an animal in a threatening situation, such as a cold environment to measure thermogenic capacity (Chappell, 1984) or into water to measure swimming endurance (Friedman *et al*., 1992; Huang *et al*., 2016). Presumably, these tests generate performance outputs that are less influenced by the animal's response to humans. Taking measurements of glucocorticoid hormones immediately after the performance tests may help determine the amount of stress generated by the forced‐exercise protocol, and this could be compared to levels observed after behavioural tests and standard restraint tests (i.e. to determine stress‐induced hormone levels). One can also use physiological measurements to attempt to verify that animals have performed maximally, such as examining blood glucose or lactate or whole‐body lactic acid levels after endurance trials that are intended to exhaust the animal (e.g. see Arnold & Bennett, 1984; Booth *et al*., 2010; Meek *et al*., 2009).

An important consideration when thinking about a behaviour–performance continuum is the concept of central fatigue. The basic idea, in (overly) simple terms, is that performance abilities may be set not by muscles fatiguing or some other ‘peripheral’ factor, but rather by the brain (a ‘central’ factor) shutting an animal down before damage occurs. For example, in principle, a cheetah chasing an antelope might experience muscle fatigue or it might begin to overheat before muscle fatigue occurs, and then the brain might shut down the animal, thus avoiding thermal damage to tissues. In reality, overheating is not why cheetahs terminate chases (Hetem *et al*., 2013).

Cheetahs aside, studies of humans have led to the proposal that ‘fatigue in any form of exercise may form part of a regulated, anticipatory response co‐ordinated in the subconscious brain, the ultimate goal of which is to preserve homeostasis in all physiological systems during exercise’ (Noakes & St Clair Gibson, 2004, p. 648). This view stems from the empirical observation that a reserve ability to recruit muscle often exists at the point of fatigue, indicating that the central nervous system is regulating performance under various test conditions. Thus, the brain, which obviously controls and causes behaviour, may also be a major determinant of locomotor performance in various tests. A thorough review of the ‘central governor model’ of fatigue is beyond our scope, but the idea remains controversial and an area of ongoing research (e.g. see Claghorn *et al*., 2017; Hopkins, 2009; Weir *et al*., 2006).

## V. FUTURE WORK

One promising way forward may be to measure multiple aspects of behaviour and performance that are differentially positioned along the behaviour–performance continuum, paired with concurrent physiological measurements (e.g. blood glucocorticoids, blood glucose, and blood, muscle or whole‐body lactic acid levels) to verify how the different protocols generate stress and motivation, as well as whether a given type of performance (e.g. maximum endurance capacity) is actually being achieved.

Preliminary studies can be crucial for performance measurements, especially with species that have not previously been studied. For example, studies of maximal sprint speed not uncommonly find that speed increases across trials, from day to day, and some have conducted trials across several days to determine how many it takes before a plateau is attained (e.g. Bennett, 1980). Similarly, based on early studies (e.g. Koch *et al*., 1998), treadmill endurance protocols with rodents typically use a couple of training (familiarisation) trials that do not attempt to achieve exhaustion, before conducting trials that try to exhaust subjects.

Such preliminary studies can also be important for attempts to measure purely behavioural traits. For example, with laboratory rodents, voluntary wheel‐running distance typically increases for approximately 2 weeks before reaching a stable plateau (e.g., see Waters *et al*., 2008), with day‐to‐day correlations (repeatability) remaining relatively consistent, even as correlations among further‐spaced days decline. If so, then which day of wheel running should be used as a behavioural measure? And should wheel running be treated as multiple traits (i.e. one for each day, e.g. Careau *et al*., 2015) or described by a curve with an intercept and slope (e.g. Biro *et al*., 2018), and perhaps quadratic term?

A related issue is that some behavioural protocols are intentionally designed to measure responses to a novel situation, and then obviously only the first test can be novel. Thus, repeated tests are by definition not measuring the same variable, even if behaviours are fairly strongly correlated from trial to trial (e.g., on open‐field behavior, see Bronikowski *et al*., 2001). Should multiple trials be conducted and, if so, how should they be analysed?

## VI. CONCLUSIONS

(1) We defined key concepts (Table 1), reviewed the literature on the links between performance and behaviour, highlighted some challenges and interesting opportunities, and proposed a behaviour–performance continuum (Fig. 1). Many potentially interesting avenues remain to be explored, both conceptually and empirically, including some that we have briefly discussed (co‐variance partitioning, motivation, and fatigue).
(2) In evolutionary biology, the term performance should be restricted to measurements made on individuals that are presumed to be maximally motivated to accomplish a pre‐determined task, which can be done in the laboratory (e.g. sprint speed on a race track) or in the wild (e.g. by chasing the animal). If a measure of speed, strength or endurance is taken on a free‐ranging animal without human intervention (e.g. as it escapes a predator, or competes with a conspecific), then the term ‘ecological performance’ applies, but in this case the distinction with behaviour starts to blur.
(3) A behaviour–performance continuum (Fig. 1) is useful for conceptualising how different protocols capture various aspects of behaviour *versus* performance, depending on the relative amount of internal *versus* external sources of motivation and the presence of choice (behaviour) or not (performance).
(4) A relatively large number of studies report statistically significant links between behavioural and performance traits, mostly at the phenotypic level, but we hope this review encourages researchers to adopt a co‐variance partitioning approach to quantify among‐individual and genetic correlations.
(5) No ‘universal’ hypothesis (i.e. based on a single underlying mechanism) can explain how behaviour ‘should’ relate to performance, with respect to either proximate or ultimate causation (Garland *et al*., 2022; Mayr, 1961).
(6) We clearly need studies that relate both behaviour and performance to Darwinian fitness measured in the wild, and particularly tests for the possibility of correlational selection (e.g. see Miles *et al*., 2007) arising from compensation processes. Mechanistic studies are also clearly needed; for example, it would be interesting to tackle the possibility of energetic trade‐offs or shared biochemical or endocrine pathways linking behaviour, performance, and even life‐history traits (e.g. see Dantzer & Swanson, 2017; Finch & Rose, 1995; Garland *et al*., 2022; Sinervo & Svensson, 1998).
(7) The co‐specialisation hypothesis remains counterintuitive in some cases, as it implies the existence of, for example, ‘slow and bold’ individuals, but manipulative studies may help identify the mechanisms involved (e.g. individual experience and predation detectability). Care must also be taken not to engage in a Panglossian reasoning (Gould & Lewontin, 1979), and non‐adaptive, proximate mechanisms (e.g. shared endocrine effects) may explain trait combinations that appear disadvantageous in all situations.
(8) Although intra‐individual variation and motivation can be seen as nuisance variables in studies of performance, they can also become part of a comprehensive study of the relative importance of variation across levels (e.g. among individuals, among tests within individuals, among trials within tests, among sections within trials).

## Supporting information


**Data S1.** R code to illustrate how to estimate the correlation between the phenotypic variation in a singly measured trait and the repeatable component of variation of another trait.

**Data S2.** Data set used to illustrate how to estimate the correlation between the phenotypic variation in a singly measured trait and the repeatable component of variation of another trait, from Courtene‐Jones & Briffa (2023).
